# Model parameterization of robotic systems through the bio-inspired optimization

**DOI:** 10.1371/journal.pone.0325168

**Published:** 2025-06-04

**Authors:** Roberto Castro-Medina, Miguel Gabriel Villarreal-Cervantes, Leonel Germán Corona-Ramírez, Geovanni Flores-Caballero, Alejandro Rodríguez-Molina, Ramón Silva-Ortigoza, Víctor Darío Cuervo-Pinto, Andrés Abraham Palma-Huerta

**Affiliations:** 1 Escuela Superior de Ingeniería Mecánica y Eléctrica - Unidad Zacatenco, Instituto Politécnico Nacional, Mexico City, Mexico; 2 Centro de Innovación y Desarrollo Tecnológico en Cómputo, Instituto Politécnico Nacional, Mexico City, Mexico; 3 Unidad Profesional Interdisciplinaria de Ingeniería y Tecnologías Avanzadas, Instituto Politécnico Nacional, Mexico City, Mexico; 4 Depto. de Ingeniería Electromecánica, Tecnológico Nacional de México/ITS del Occidente del Estado de Hidalgo, Mixquiahuala de Juárez, Hidalgo, Mexico; 5 Colegio de Ciencia y Tecnología, Universidad Autónoma de la Ciudad de México, Mexico City, Mexico; Indian Institute of Technology Patna, INDIA

## Abstract

The accurate modeling of dynamic systems, particularly robotic ones, is crucial in the industry. It enables simulation-based approaches that facilitate various tasks without requiring the physical system, thereby reducing risks and costs. These approaches range from model-in-the-loop (MiL), where a simulated model of the real plant is used for controller design, to hardware-in-the-loop (HiL), which provides more realistic simulations on specialized real-time hardware. Among these, MiL is widely adopted due to its simplicity and effectiveness in developing control strategies. However, to fully leverage the advantages of MiL, developing a robust and accurate system model parameterization methodology is essential. This methodology should be adaptable to a wide range of applications, adopt a holistic approach, and balance the cost-benefit trade-offs in model characteristics. Achieving this, however, introduces additional challenges related to system complexity and the inherent properties of the model. To address these challenges, this work proposes a model parameterization approach for robotic systems using bio-inspired optimization to develop accurate and practical models for system design. The approach formulates an optimization problem to determine the dynamic model parameters of a robot, ensuring its behavior closely resembles that of the real system. Due to the complexity of this problem, bio-inspired optimization techniques are particularly well-suited. The proposed method is validated using a theoretical, non-conservative model of a three-degree-of-freedom serial robot. The dynamic parameters of its three links were identified to effectively generalize the real system. To solve the optimization problem, three bio-inspired algorithms were employed: the genetic algorithm, particle swarm optimization, and differential evolution. The optimal parameterization obtained for the robot model demonstrated the effectiveness of the proposed approach in a MiL simulation environment, achieving an overall correlation of 0.9019 in the experiments. This correlation highlights the model’s ability to predict the robot’s behavior accurately. Additionally, the methodology’s efficacy was further validated in another electromechanical system, the reaction force-sensing series elastic actuator, yielding a correlation of 0.8379 in the resulting model.

## Introduction

Competition among industrial sectors generates new production and control system techniques that maximize processes’ efficiency and flexibility.

Developing a control strategy for an automatic system requires physical prototyping, which can be time-consuming and expensive, especially when testing edge cases [1–3]. These tests can push the systems to unsafe conditions and may result in loss of material and equipment, among other expenses and risks. To overcome these difficulties, it is often desirable to rely on the use of simulation technology. Depending on the scope, the simulation environment, and the final purpose, simulation techniques or approaches can be classified into:

1. Model-in-the-loop (MiL) [4]: The control logic is simulated using mathematical models of the system and its environment.
2. Software-in-the-loop (SiL) [5]: The controller is implemented within simulation software that mimics the system’s behavior and its environment.
3. Processor-in-the-loop (PiL) [6]: The control strategy is executed on the actual hardware or processor to govern the behavior of a system within a simulated environment.
4. Hardware-in-the-loop (HiL) [7]: Hardware elements are used to simulate the system, the control strategy, and the operating environment.
5. System-in-the-loop (XiL) [8]: Integrates all components of the system (hardware and software) into the simulation for thorough testing and validation of the entire system.

Particularly, MiL offers various advantages: it is low-cost, fast to develop, can detect early problems with control strategies and system models, and is flexible and scalable in terms of the variety of system configurations and simulation conditions that can be imposed on the environment. It is crucial to study, generate, or utilize methods that generate models that closely mimic the real response of a system to develop the MiL approach properly.

System identification encompasses various techniques, approaches, and methodologies to obtain models of dynamic systems from information estimated or acquired from their inputs and outputs. The identification process typically includes four main stages [9]:

- Data acquisition: Where the input and output variables that best represent the behavior of the dynamic system are measured or estimated. This measurement or estimation can be performed in the time or frequency domain for this type of system using sensors or transducers.
- Selection of a model structure: Here, a mathematical structure is derived that can relate the values of the system inputs to their corresponding outputs. Such structures can be simple algebraic relations, sets of differential equations, or more sophisticated computational models.
- Model structure tuning: Regardless of their type, model structures have several adjustable parameters on which the accuracy of relating the inputs to the system’s outputs depends on a correct settling. If the parameters are set properly, the model will be able to mimic the behavior to a large extent. On the other hand, a wrong adjustment of these parameters can trigger a behavior of the model very different from that of the real system.
- Evaluation of the adjusted model: This last stage verifies that the adjusted model obtained is adequate to satisfy the application’s needs.

The four stages necessary to identify dynamic systems described above can be performed online or offline. In the offline approach, information on the system’s inputs and outputs is acquired at the beginning of the identification process, ensuring that the data are representative and sufficient and assuming that the system’s behavior will remain unchanged. Subsequently, the remaining three stages are carried out, and the validated model remains invariant and is used for some practical purposes [10], e.g., for the offline tuning of a control system [11]. On the other hand, online identification is a process that is carried out considering that the behavior of the dynamic system will change in the short term. Therefore, the four stages are repeated at certain time intervals during the operation of the dynamic system. One of the most common uses of this identification type is in the indirect adaptive tuning of controllers [12].

At present, different methods implement the above four stages to obtain adequate models of systems. Therefore, identification methods can be classified according to the type of information used to derive an accurate model of a system [13]. In this sense, there are time-domain methods, where information obtained from sensors in the system can be used directly during the identification process [14]. On the other hand, in frequency-based methods, the information from the sensors is not used directly but is converted to the frequency domain [15,16]. Parametric or non-parametric identification methods can be found depending on the model structure. Parametric methods search for a specific model structure’s parameter values that describe the system’s dynamic behavior [17,18]. On the other hand, non-parametric methods do not use a specific model structure but instead occupy generalized mathematical or computational structures with parameters that must be adjusted to replicate a particular system behavior [19,20]. System identification can be performed using state-space methods, which model a system’s behavior through its internal states with a minimal set of variables [21]. Alternatively, non-state-space methods focus only on input-output relationships to derive a model without accounting for internal dynamics [22].

Different models are frequently used among the identification approaches presented up to this point. Representative examples include linear [23] and nonlinear [24] autoregressive models; linear [25] and nonlinear [26] state-space models; transfer function models [27]; Gaussian regression processes [28]; and neural networks [29]. Now, concerning estimation algorithms, some common choices include least squares algorithms [30], classical optimization techniques [31], machine learning training methods [32], and metaheuristics [33].

The choice of a method, model, and estimator in system identification depends on several factors, including the nature of the studied dynamic system (e.g., whether it is complex in terms of its inputs and outputs, whether it can be described using established rules, whether it is subject to change, etc.), the characteristics of the available data (e.g., whether all input and output variables can be measured or estimated, whether the amount of information is sufficient and relevant, etc.), and the specific objectives of the analysis (i.e., the ultimate use of the identified model, e.g., for controller tuning or virtual simulation, etc.).

System identification is especially relevant for deriving models of robotic systems. A robotic system can be defined as a collection of mechanical elements interconnected through joints that, when properly planned and controlled, allow very complex movements or tasks. These systems are growing in importance every day due to their ability to accurately perform automated tasks in different application areas ranging from healthcare [34] to space exploration [35].

In the context of robotic manipulators, many factors that can help select methods, models, and estimators for identification are evident. In the first instance, robotic manipulators possess nonlinear (or highly nonlinear) behaviors that can grow in complexity depending on their degrees of freedom. Since these systems are a subset of mechanical systems, it is possible to derive accurate model structures using the axioms of classical mechanics, i.e., Newton’s laws. These structures are naturally found in the state space and time domain and are parameterized by physical variables such as mass, inertia, friction, etc. The states used to describe the configuration of robotic systems at any instant include the positions and velocities of each of their joints, regardless of their type. Likewise, the inputs of this type of system are related to the amount of energy that is included in their motion-generating elements, i.e., in their actuators. In addition, the information on the inputs and outputs, related to the system states and energy inputs, can be acquired relatively simply using transducers or estimated using observers. Due to the inherent complexity of such systems, the identification problems are expected to be equally complex, so the use of metaheuristic techniques may be necessary.

**Fig 1:**
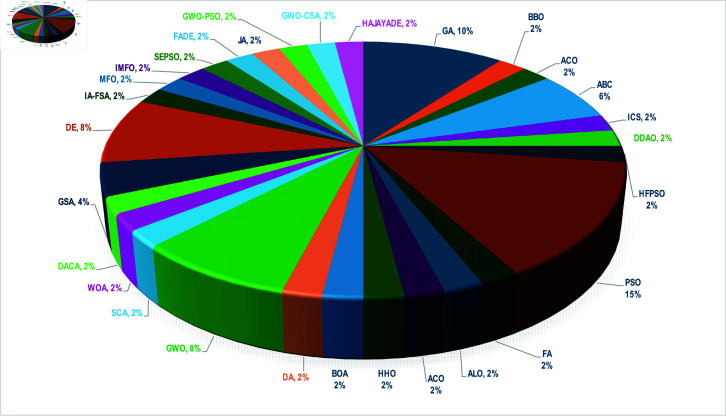
Percentage of times that metaheuristic algorithms are used in the parameter estimation of dynamic systems.

Table 1 provides a bibliographic review of recent studies that utilize metaheuristic optimization (Met. Opt.) for estimating model parameters in dynamic systems. This table reveals the frequent use of the parameter estimation in robotic applications, with 50% of the research, followed by the application in the actuators at 30%, and in other mechanical and electrical systems such as phase inverter circuits and mass-spring-damper systems at 20% of the reported works. Fig 1 aids in graphically depicting the algorithms utilized in the studies referenced in Table 1. It can be observed in Fig 1 that particle swarm optimization (PSO) is used in 15% of the studies, followed by the genetic algorithm (GA) at 10%, differential evolution (DE) algorithm and grey wolf optimization (GWO) at 8%. Algorithms such as the artificial bee colony (ABC) and gravitational search algorithm (GSA) accounted for 6% until 4% of the studies. Other algorithms represented 2% collectively in this review.

**Table 1:** Works related to the model’s parameter estimation by Met. Opt.

Ref.	Year	Platform	Used algorithm(s)*	Obtained parameters	Description
[36]	2024	Parallel robot (spacecraft docking simulation system)	Improved PSO	The initial lengths of the six hydraulic cylinders, the position coordinates of the twelve Hooke’s joints	A kinematic parameter identification method based on forward kinematics is presented for a parallel robot in a docking motion simulation system. This method utilizes an improved PSO algorithm to accurately identify and correct kinematic parameter errors of the parallel robot.
[37]	2024	Solar photovoltaic system panel (PV)	ANN [38], PSO [39]	Current source by incident light, reverse saturation current, resistance in time series, capacitance in time series, total serial resistance, total voltage	The initial population of particles is selected based on the tuning results of the ANN model parameter range classifier (MPRC), providing solutions close to the optimum. The PSO algorithm then begins its search, obtaining acceptable solutions within a few iterations.
[40]	2023	Clamped-clamped nonlinear two-degrees-of-freedom system (two masses)	PSO, GA [41]	Viscous damping coefficients, linear stiffness, Cubic stiffness coefficients, mass	A two-mass system with two supports was used to test the effectiveness of GA and PSO algorithms for motion prediction under excitation. The results show that while the PSO method significantly increases accuracy, it demands high computational resources. Conversely, the GA requires similar computational effort but fails to provide accurate solutions.
[42]	2023	6-DOF UR5 robot manipulator	CSA [43], PSO, GWO [44], GWO-PSO [45], GWO-CSA	The components of inertia tensor, static friction, viscous friction, coulomb friction, Stribeck velocity, Stribeck shape factor	The inertia parameters and coefficients of the Coulomb viscous friction model, Stribeck model, and centrosymmetric static friction model (CSFM) for all joints of the UR5 robot are identified using a hybrid grey wolf optimizer and particle swarm optimization (GWO-PSO) procedure. The identified model is tested with five random trajectories in the task space and a finite Fourier series in the joint space. Finally, the performance of the models produced by different algorithms is compared.
[46]	2022	Permanent Magnet DC Motor	GWO, JA [47], CSA	Armature resistance, Armature self-inductance, Moment of inertia, Friction coefficient	A comparison of the experimental responses of a motor stimulated with step signals using three different metaheuristic techniques is conducted.
[48]	2022	Photovoltaic (PV) system	HAJAYADE	Current from the solar cell, reverse saturation current, feature factor of the diode, total serial resistance	The HAJAYADE algorithm (Adaptive Jaya + DE Rank/Best/1 mutation) is employed for parameter identification of PV systems using the most common mathematical models: the single diode (SDM) and the double diode (DDM).
[49]	2021	Mass-spring-damper system	GA, BBO [50], ACO [51], ABC [52]	Mass parameters, damping coefficients and spring deformation	Three case studies are analyzed to obtain system parameters under various conditions, including data disposition and noise. The applied metaheuristic algorithms prove to be effective alternatives for parameter identification in mechanical systems.
[53]	2021	Permanent Magnet DC Motor	CSA	Winding resistance, winding inductance, rotor moment of inertia, viscous friction coefficient	Steady-state relationships are incorporated into the cuckoo search algorithm (CSA) to obtain system parameters, creating an improved version. This enhanced CSA is compared with the original CSA and the Steiglitz-McBride method.
[54]	2020	Planning of restricted paths in robots (numerical simulation)	DDAO, HFPSO [55], PSO, FA [56], ALO [57], HHO [58], BOA [59], DA [59], GWO, SCA [60], WOA [61], DACA [62]	Coefficients of the polynomial of a spline curve	The performance of a proposed algorithm, based on simulated annealing improved with random search operators (DDAO), is analyzed for path planning with obstacles in a mobile robot’s workspace and compared with multiple algorithms.
[63]Ch. 2	2020	Induction motor	GSA [64], DE [65], PSO, ABC	Stator resistance, rotor resistance, stator reactance, motor slip	Parameter estimation of an induction motor is performed using the gravitational search algorithm (GSA) with both exact and approximate circuit models. The performance of GSA is also compared to other bio-inspired algorithms.
[63]Ch. 3	2020	Induction motor	ICSA, DE, ABC, GSA	Stator resistance, rotor resistance, stator reactance, motor slip	Parameters of an induction motor are obtained by modifying the crow search algorithm (CS [66]) with a dynamic probability knowledge operator (DAP). The obtained parameters are used to analyze power motor losses in kW per year. The modified CS algorithm’s (ICSA) performance is compared with other algorithms in the literature.
[67]	2020	Generic manipulator robot (6 d.o.f in numerical simulation)	IA-FSA	Masses, moments of inertia in the fifth and sixth links, frictions, lengths to centers of mass	The combined connection method is used to divide the robot into two parts: one analyzing links 1 to 5 and the other focusing on the sixth link. Additionally, the artificial fish swarm algorithm (FSA [68]) is implemented with an artificial vision enhancement to improve the field of view of solutions, along with other mechanisms.
[69]	2019	Phase inverter circuit	IMFO, PSO, GA, GWO, MFO [70]	Inductance, capacitance and resistance of the circuit	The moth-to-fire search algorithm (IMFO) is improved to search for circuit parameters, with its results compared against other bio-inspired algorithms.
[71]	2018	SCARA type robot (3 d.o.f. in numerical simulation)	GA, curve fitting methods	Masses, moments of inertia in z-axis	This work reviews the parameter identification procedure for a robot, comparing different methods: the method of least squares, Adaline networks, extended Kalman filters, and genetic algorithms.
[72]	2016	Planar manipulator robot (2 d.o.f. in numerical simulation)	SEPSO, SPSO, GA	Masses, moments of inertia, length to the center of mass, and centroid angle	A modification of the PSO algorithm, incorporating the socio-emotional model (SEPSO), is proposed. Its performance in obtaining parameters is compared with the standard PSO algorithm and the genetic algorithm (GA).
[73]	2014	CRS A456 manipulator robot (6 d.o.f.)	FADE, DE, OLS	Base link parameters; moment of inertia, coefficient of viscous friction, and the positive and negative Coulomb coefficients of friction	The performance of the fuzzy/adaptive DE estimation (FADE) technique is compared to the traditional DE algorithm and the ordinary least squares (OLS) technique. FADE demonstrates superior computational performance.

ANN (artificial neural networks), CSA (Cuckoo Search Algorithm), CS (Crow Search Algorithm), JA (Jaya Algorithm), HAJAYADE (Hibrid Adaptative Jaya/Diferencial Evolution Algorithm), DE (Diferencial Evolution), GA (genetic algorithm), PSO (Particle Swarm Optimization), TAPSO (Triple Archive Particle Swarm Optimization), BBO (Biogeography-Based Optimization Algorithm), ACO (Ant Colony Optimization Algorithm), ABC (Artificial Bee Colony Algorithm), DDAO (Dynamic Differential Annealed Optimization), HFPSO (Hybrid Firefly and Particle Swarm Optimization algoritm), ALO (Ant Lion Optimizer algorithm), HHO (Harris Hawks Optimization algorithm), BOA (Butterfly Optimization Algorithm), DA (Dragonfly Algorithm), GWO (Grey Wolf Optimizer), SCA (Sine Cosine Algorithm), WOA (Whale Optimization Algorithm), DACA (Dynamic Advanced Clustering Algorithm for Sensor Networks), ICSA (Improved Crow Search Algorithm), IA-FSA (Improved Artificial Fish Swarm Algorithm), MFO (Moth Flame Optimization algorithm), IMFO (Improved Moth Flame Optimization algorithm), SEPSO (Social Emotional PSO), SPSO (Social PSO), GSA (Gravitational Search Algorithm), FA (Firefly Algorithm), FADE (Fuzzy Adaptative DE Algorithm).

The limitations identified in the reviewed literature, summarized in Table 1, highlight that system model parameterizations are often tailored to specific applications or individual system components. This approach typically characterizes system dynamics within a restricted movement space or approximates the system using a linear model. As a result, the models tend to overfit the specific task or the assumed linear representation, limiting their generalization ability. It is important to mention that a good generalization is essential for MiL simulations. Another key limitation is that many reviewed studies focus on a single stage of the overall modeling process within a specific domain, aiming to enhance the parameterized model while overlooking other influential factors. For instance, some studies concentrate solely on algorithm development to refine model parameters but neglect other process stages that could further improve model accuracy. Similarly, others apply only one type of optimization algorithm, failing to explore alternative metaheuristic approaches that might yield better parameterization results. Additionally, the reviewed studies often lack flexibility in balancing trade-offs between optimization criteria. In many cases, this trade-off is determined arbitrarily, leading to a parameterization that prioritizes one objective at the expense of others. This limitation restricts the ability to explore more balanced solutions that could enhance overall model performance. Ultimately, these limitations suggest that existing studies do not provide models well-suited for MiL simulation environments. Moreover, a comprehensive, step-by-step methodology for optimizing robotic system parameters using metaheuristic techniques—along with its practical application in Model-in-the-Loop (MiL) testing, is noticeably absent from the literature.

Motivated by these limitations and gaps, the main contributions of the paper are listed below:

- Proposal of the model-based parametric bio-inspired optimization (MbPBO) methodology: This work introduces an offline bio-inspired optimization-based model parameterization methodology for robotic systems, termed MbPBO. The MbPBO is designed for Model-in-the-Loop (MiL) testing and provides a structured approach to achieve accurate parameterization. This methodology significantly improves the alignment between simulation and real-world experimentation.
- Guidelines for MiL implementation: The MbPBO methodology offers a practical framework for researchers and practitioners looking to implement MiL test systems using bio-inspired optimization. It outlines essential steps and considerations to ensure effective model parameterization.
- Validation through case studies: The application of MbPBO in two case studies demonstrates the quality, consistency, and adaptability of the obtained models. These case studies highlight the model’s strong correlation with real experimental data and its potential for broader application in MiL test environments. Specifically, the methodology is implemented for a three-degree-of-freedom serial robot and a reaction force-sensing series elastic actuator, validating its flexibility and effectiveness across different electromechanical systems.

The rest of this paper details the proposed MbPBO approach for robotic systems based on bio-inspired optimization. It also describes the implementation of the proposed approach on an RRR robot and an electromechanical system, the reaction force-sensing series elastic actuator, to show its scope. The discussions and conclusions of the proposal are drawn at the end of the paper.

## Model-based parametric bio-inspired optimization methodology

**Fig 2:**
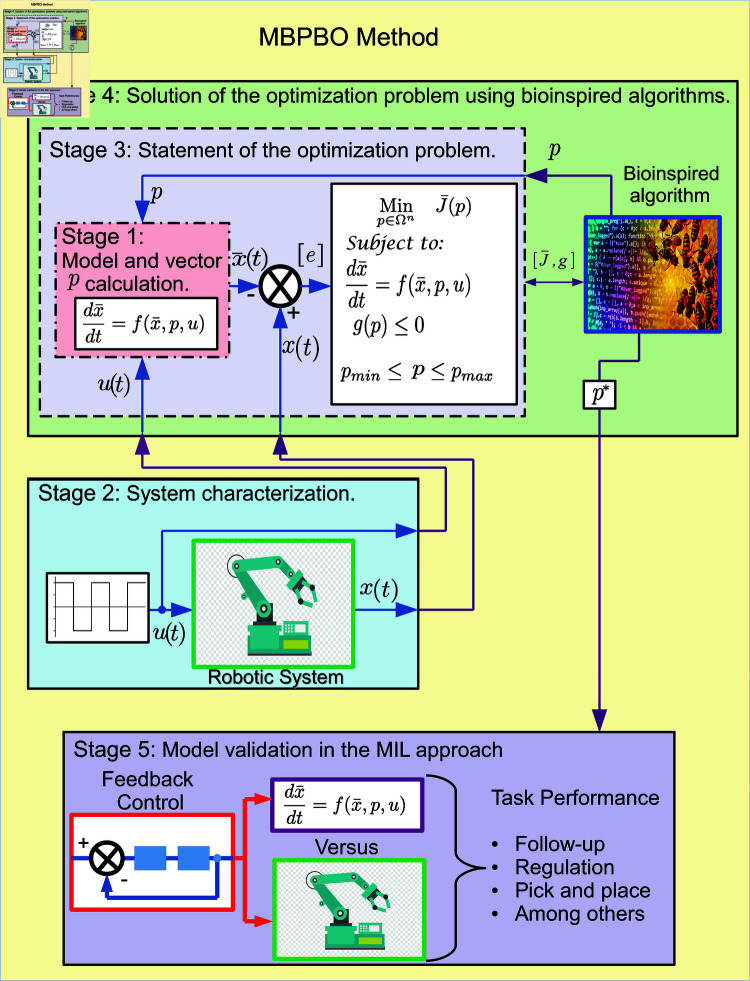
General scheme of the MbPBO methodology.

The current section presents the proposed model-based parametric bio-inspired optimization (MbPBO) methodology. This methodology allows the dynamic behavior of systems to be identified based on bio-inspired optimization and then validated using the MiL approach. The proposed MbPBO methodology is observed in Fig 2. This figure shows the stages considered in the methodology, landed towards an application for any robotic system.

The following subsections describe the stages that conform to the proposed MbPBO methodology.

### Stage I: Determination of the base model and its key parameters

In this stage, the base model of the MbPBO methodology is defined. A white-box model is required, as it incorporates the mathematical relationships governing the system’s physical behavior. Black-box models are excluded because they obscure or eliminate these relationships, which are crucial for validation in the MiL stage. Additionally, black-box models tend to be less interpretable and are typically valid only within a limited workspace region.

For mechanical systems, the direct dynamic model [74] is commonly used, as it is suitable for control and numerical simulation. In robotic systems, this model is typically represented by nonlinear differential equations and is preferably expressed in state-space form.

Three aspects are considered at this stage: (i) the selection of parameters to be estimated, (ii) the initial values proposed for these parameters, and (iii) the reduction of the model by eliminating dynamics that do not substantially influence the system’s behavior. These aspects are discussed in detail below.

#### (i) Selection of the parameters to be estimated

The model’s parameters define its behavior and must be carefully identified to achieve an accurate representation. These parameters primarily determine the system’s response to external stimuli. Since models are tailored to specific system properties, mathematically similar dynamic models, such as the closed-form dynamics of a robotic manipulator, can exist. However, the specific parameter values differentiate one system from another.

In this stage, the *n* parameters to be identified are defined, forming the design variable vector $p\in {\mathbb{R}}^{n}$, which will be used in subsequent stages of the MbPBO methodology.

#### (ii) Initial values proposed for the parameters

As discussed later, the estimation techniques in the MbPBO methodology rely on metaheuristic algorithms, which typically begin their search with randomly initialized values for the design variables in *p*. However, in some cases, selecting appropriate initial values can enhance the final results. These values should be chosen based on estimates that are close to the real ones. For existing systems, it is recommended to use manufacturer-provided values as a starting point. When such data is unavailable or for entirely theoretical systems, computer-aided design (CAD) tools can be used to approximate key aspects, such as component dimensions and material properties. In the case of non-conservative models, parameters such as friction and hardness are often not provided by manufacturers or specified in CAD programs. Instead, these values can be estimated based on theoretical knowledge, ensuring the model remains solvable and well-defined. For example, the coefficient of dry friction varies depending on material type (e.g., metals, plastics), with accepted ranges documented in specialized literature [75], which can serve as a reference.

#### (iii) Model reduction

In robotic systems, the complexity of the model and the number of required computations generally increase with the number of degrees of freedom (d.o.f.) [74]. Due to this complexity, analytical solutions are often impractical, necessitating the use of numerical methods and computational resources. Since the model is intended for simulation, it is crucial to account for the computational limitations of the simulator. Model complexity is determined by the computational resources required for accurate interpretation [76], often measured by the number of operations involved. To ensure efficient simulation execution, model simplification is necessary. This can be achieved by precomputing frequently recurring terms and applying mathematical properties to reduce redundant expressions. Simplification helps maintain model validity within the experimental framework while optimizing computational performance. Additionally, the design variable vector is updated accordingly.

### Stage II: Characterization of the system’s behavior

In [77], various methods for obtaining system parameters are discussed. One approach involves direct physical measurement, which may require partial disassembly of components, making it impractical in some cases. Another option is to use manufacturer-provided values or computer-aided design (CAD) programs, though inaccuracies in manufacturing and assembly can complicate parameter estimation. A third and often more effective alternative for MiL applications is parametric identification, where parameters are inferred indirectly from the system’s dynamic behavior.

At this stage, experimental data at different excitation frequencies are collected for use in model optimization. In MiL testing, system parameters are assumed to remain constant over time, so data collection is performed offline.

The experiment involves extracting system response data under external excitation [76]. A key principle in system parameter characterization is the application of a persistent excitation signal [78], which induces a response that exhibits diverse dynamic behaviors. As highlighted in [79], such a signal provides sufficient *a priori* information about the system, improving parameter identification accuracy and robustness against measurement noise.

To generate the excitation signal, [77] suggests using a control signal derived from an optimized trajectory, particularly for robotic manipulators. However, this approach results in parameters specific to the given trajectory, limiting generalization to other tasks. In this study, a sinusoidal or square wave signal with variable periods and bounded amplitude is used as the excitation signal, denoted as *ref*(*t*).

Experimental system response data are collected based on the applied excitation signal *ref*(*t*). The number of recorded measurements depends on available resources, with accuracy closely tied to the quality of the measurement tools. In robotic systems, state variables ($x\in {\mathbb{R}}^{m}$) evolve over time and represent quantities such as position ($q\in {\mathbb{R}}^{o}$), velocity ($\dot{q}\in {\mathbb{R}}^{o}$), acceleration ($\ddot{q}\in {\mathbb{R}}^{o}$), among others.

In electromechanical systems, excitation signals are typically controllable system variables, such as voltage. If a cascade control architecture is used, these signals correspond to the outer-loop reference signals of the system, indirectly modifying input variables and exciting system dynamics.

Once collected, the experimental data will be utilized in the next stage of the methodology.

### Stage III: Establishment of a mathematical programming problem

The parameters *p* are obtained by solving an optimization problem, which is formulated in this stage of the methodology.

Once the experimental data of the system’s state variables (*x*) have been collected (Stage II) and the theoretical model has been defined (Stage I), the same excitation signal *u*(*t*) is applied to the model using a given vector of design variables *p*. The state error vector $\tilde{e}\in {\mathbb{R}}^{m}$ is then computed as the difference between the model’s estimated states and the system’s directly measured states over the experiment’s duration. Through a closed-loop, iterative identification process, the objective is to determine a vector p that minimizes $\tilde{e}$.

In the MbPBO methodology, each objective function typically represents a state error variable. If multiple state errors share the same units, a weighted sum of these errors can also be used as an objective function. To ensure convexity, the methodology minimizes the squared state error, as defined in Eq. (1), where $n_{J_{i}}$ represents the number of state error elements in the *i*–*th* objective function (for a single error, $n_{J_{i}}=1$).

$$J_{i}(p)=\sum_{\bar{i}=1}^{n_{J_{i}}}\int_{0}^{t_{f}}{\tilde{e}}_{\bar{i}}^{T}{\tilde{e}}_{\bar{i}}dt$$ (1)

The optimization problem can be formulated using a multi-objective approach [80], expressed in equations (2) to (6), where $J(p)\in {\mathbb{R}}^{n_{J}}$ represents the vector of objective functions, $p\in {\mathbb{R}}^{n}$ is the vector of design variables and $g\in {\mathbb{R}}^{n_{g}}$ and $h\in {\mathbb{R}}^{n_{h}}$ are the inequality and equality constraints that limits or provides precise conditions to the problem, respectively. Here, $\Omega \in {\mathbb{R}}^{n}$ denotes the space of feasible solutions. Additionally, $\bar{x}$ represents the set of states of the system model, where equation (3) describes its dynamics, and $p_{min},p_{max}$ establish the minimum and maximum limits of the vector *p*, respectively.

$$\underset{p\in \Omega }{\text{Min}}J(p)=[J_{1}(p),J_{2}(p),...,J_{n_{J}}(p)]$$ (2)

subject to:

$$\frac{d\bar{x}}{dt}=f(\bar{x},p,u)$$ (3)

$$g_{j}(p)\leq 0,j=1,2,...,n_{g}$$ (4)

$$h_{k}(p)=0,k=1,2,...,n_{h}$$ (5)

$$p_{min}\leq p\leq p_{max}$$ (6)

In the MbPBO methodology, the multi-objective optimization problem is reformulated as a mono-objective optimization problem through the weighted sum approach [81]. In the weighted sum approach, the multi-objective optimization problem is converted into a single-objective problem by assigning a weight to each objective function included in the vector *J* (2). The objective function is then expressed as a weighted sum of all individual objectives. Adjusting the weights allows for exploring different trade-offs between objectives, allowing for a better understanding of the Pareto-optimal solutions. So, the single-objective function $\bar{J}$ in (7) is the combination of *n*_*J*_ elements in the vector *J*, each expressed in (1) and weighted by $\mu_{i}\in R$. The *i*–*th* weight $\mu_{i}$ sets the priority (importance) in terms of the objective function for the optimization process, i.e., higher values indicate more preference in the minimization of the *i*–*th* term *J*_*i*_ in the single-objective function $\bar{J}$. A normalization process in terms of the objective function (*J*_*i*_) is required to ensure dimensionless values within the range [0,1] and to set the condition for the weight values as $\sum_{i=1}^{n_{J}}w_{i}=1$ [81]. The normalization is performed by dividing the *i*–*th* term *J*_*i*_ by its maximum value $J_{i_{MAX}}$. The maximum value is determined by evaluating *J*_*i*_ using the design variable vector obtained when all other terms are minimized individually.

$$\bar{J}(p)=\sum_{i=1}^{n_{J}}\mu_{i}\frac{J_{i}(p)}{J_{i_{MAX}}}$$ (7)

Typically, the optimization problem in this stage can be formulated as shown in equations (8) to (12). The objective is to find the vector of design variables *p* that minimizes the weighted objective function $\bar{J}$ in (7), subject to the system model’s dynamic behavior (9), inequality constraints (10), equality constraints (11), and limits on the design variables (12).

$$\underset{p\in \Omega^{n}}{\text{Min}}\bar{J}(p)$$ (8)

subject to:

$$\frac{d\bar{x}}{dt}=f(\bar{x},p,u)$$ (9)

$$g_{j}(p)\leq 0,j=1,2,...,n_{g}$$ (10)

$$h_{k}(p)=0,k=1,2,...,n_{h}$$ (11)

$$p_{min}\leq p_{i}\leq p_{max}$$ (12)

### Stage IV: Solving the optimization problem using bio-inspired algorithms

In this stage, the optimization problem formulated in Stage III is solved. To align the parameter vector with the experimental system’s behavior, the problem, originally defined in continuous time, is transformed into a discrete-time formulation using the transcription method [82]. This transformation enables the effective application of optimization techniques. The system’s governing differential equations, which serve as inherent constraints in the optimization problem, are discretized into finite states using numerical integration methods such as Euler’s method or Runge-Kutta methods. This approach ensures that dynamic constraints are satisfied simultaneously during the optimization process. Once the transcription is complete, the following steps are carried out:

#### (i) Trade-off selection

Since the multi-objective problem has been transformed into a single-objective optimization problem using the weighted sum approach, it is crucial to establish a desired *a priori* preference among the terms in the objective function to improve the obtained model in the methodology. To achieve this, different weight configurations (μ) are tested, each yielding a solution that reflects a specific trade-off in the weighted objective function. To determine the best trade-offs, the Pareto front is constructed by evaluating Pareto optimality [83] across all solutions obtained from different weight configurations. The identified Pareto-optimal solutions represent the most balanced trade-offs among the objective function terms. Finally, based on the resulting Pareto front, the designer can select the most suitable trade-off for the specific application.

#### (ii) Algorithm selection

The proposed MbPBO methodology utilizes bio-inspired algorithms for system parameter estimation. These algorithms offer several advantages, including the ability to handle complex problems with reasonable computational costs, support for parallelization, ease of implementation, and adaptability to diverse contexts [84].

It is recommended to begin by selecting algorithms commonly used in the literature (see Table 1). The GA, PSO, and DE were chosen for analysis in this study. These algorithms are widely used due to their adaptability, population-based search strategies, robust performance, and ease of implementation [85–87]. Their effectiveness in both research and real-world applications has contributed to their growing popularity in optimization problems.

#### (iii) Performance analysis of bio-inspired algorithms

Selecting the appropriate optimization technique based on empirical evidence is crucial for obtaining the best model parameters. Since no single algorithm outperforms all others across all problem types, an idea formalized by the “no free lunch theorem" [88], the MbPBO methodology recommends conducting a comparative study to evaluate the performance of representative algorithms.

The comparative study involves running each selected algorithm independently with the same number of objective function evaluations. The best solution from each execution is stored, forming a statistical sample for comparison. To ensure robust statistical representation, thirty independent runs are recommended [89]. Additionally, algorithm parameters should be fine-tuned systematically through manual adjustment (“by hand"), informed by state-of-the-art practices “by analogy"), or optimized using automated parameter tuning methods [90].

Once the sample results are collected, statistical analysis is applied to determine which optimization technique achieves the lowest objective function value, indicating minimal error between the model’s estimated states and the plant’s measured states. Comparing algorithm performance is essential for selecting the best parameter set for the system. If significant differences are observed in the solutions obtained by different algorithms, it may be necessary to enhance algorithm operators or incorporate state-of-the-art modifications to improve search efficiency.

Upon completing this stage, the optimized parameter vector p* is integrated into the system model, preparing it for validation in an open-loop control configuration and model-in-the-loop (MiL) simulation.

### Stage V: Validation of the obtained model and its use in the MiL simulation

At this stage, the model undergoes validation by integrating the optimized parameter vector p* obtained in the previous section. This process consists of two key steps.

The first step assesses whether the system model, with p*, exhibits behavior similar to the plant’s actual states when subjected to the excitation signal from Stage II. This is conducted in an open-loop control configuration. To quantify the model’s accuracy, a correlation measure (e.g., Pearson correlation coefficient) is recommended to evaluate the alignment between the model’s predicted states and the plant’s actual behavior.

The second step evaluates the parameterized model within various MiL simulation environments. MiL validation involves a series of tests conducted in the early phases of model-based design to verify model behavior and performance. These tests are performed in a closed-loop control configuration within a simulation environment such as MATLAB/Simulink, assessing predictive accuracy for specific tasks [91].

Unlike software-in-the-loop (SiL), which focuses on control programming in a specific language, or hardware-in-the-loop (HiL), which evaluates component response times and communication delays, MiL validation is primarily concerned with verifying the model’s accuracy and performance before further implementation. Early detection of logical errors at this stage significantly reduces costs and improves model quality. Additionally, the model-based design allows for adaptability to evolving requirements, ensuring alignment with end-user needs [92].

The MbPBO methodology is designed for versatile applications, allowing for the simulation of tasks such as trajectory tracking and regulation, depending on the designer’s objectives. When the physical plant is available, the most effective validation involves deploying the model in a real-world task and comparing its performance against actual system execution [93].

By the end of this stage, the validated model should closely approximate the plant’s operational behavior, facilitating subsequent phases of the model-based design cycle (SiL to HiL). MiL validation is especially critical for applications where direct testing on the physical system may pose risks. Numerical simulations with a validated model provide valuable insights into expected system behavior under various conditions.

Testbed platforms are available in the case studies presented below, allowing direct validation using real systems. The Pearson correlation coefficient is used as a metric to quantify the relationship between the model’s predictions and the actual system behavior across different test scenarios.

## Case study of the MbPBO methodology: Modeling of an RRR robot

**Fig 3:**
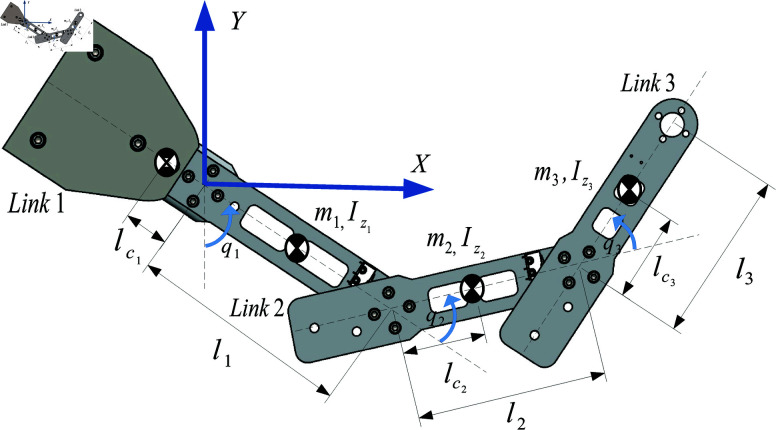
Schematic diagram of the serial robot with three degrees of freedom (d.o.f.).

A representative case study of robotic system is proposed to verify the effectiveness of the MbPBO methodology. In this case, a planar RRR manipulator robot is selected. This robot consists of three rigid links connected by rotational joints that allow relative movement between them. It is equipped with sensors to obtain the relative position of the links and actuators (motors) to enable their movement. These components allow the robot to position its end-effector, on a two-dimensional plane defined by the coordinates $[X_{w},Y_{w}]\in {\mathbb{R}}^{2}$. The manipulator is depicted in the schematic diagram in Fig 3, where the position vector q=[q_1_,q_2_,q_3_] $\in {\mathbb{R}}^{3}$ represents the rotational movements in each link, defining its generalized coordinates. Similarly, $\dot{q}=[{\dot{q}}_{1},{\dot{q}}_{2},{\dot{q}}_{3}{]}^{T}\in {\mathbb{R}}^{3}$ represents the velocity vector. The dynamic parameters of the i-th link, which are the subject of experimental identification, include the mass *m*_*i*_, the distance from the axis of movement to the center of mass $l_{c_{i}}$, the moment of inertia $I_{z_{i}}$, and the length of the link *l*_*i*_, with i=1,2,3.

This system was chosen because it has an open architecture that enables the implementation of different control strategies and tasks that are useful in the validation stage.

**Fig 4:**
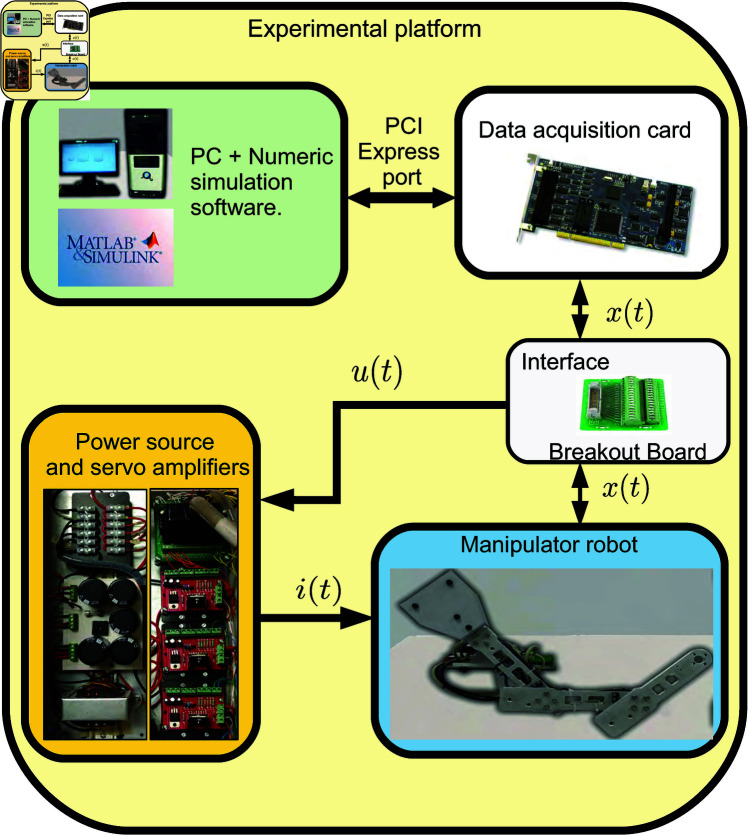
Component diagram of the experimental platform of the RRR robot.

The experimental platform of the RRR robot, shown in the diagram in Fig 4, consists of a custom-designed and manufactured non-commercial robot with three degrees of freedom. It includes three DC motors (RE-35 from Maxon Motor), their respective gearboxes, and rotary encoders (MR-256 from Maxon Motor). The DC motors are driving by three servo amplifiers LMD18245 configured in the current mode configuration (torque mode configuration). The current mode configuration incorporates a proportional-integral (PI) controller to regulate the armature current of the robot’s motors. So, a cascade control strategy is set up to govern the robot behavior, where the inner loop contains the PI current controller and the outer loop controller provides the current reference to the inner loop one. The current reference, in turn, modifies the system’s input variable (voltage), which excites the system dynamics and the users provide this reference signal. The power supply for the robot is also included, providing a 30V, 6A DC voltage source.

The outer loop controller programming has been carried out on a commercial PC (Intel Core 2 Quad Q8400 at 2.66 GHz with 4 GB of RAM), and data acquisition from the robot is performed through a Sensoray 626 card, which is adapted to one of the PCI ports of the computer system. So, in the open-loop control operation, the outer loop is made by injecting the reference signal (current reference) without feedback into the cascade system. In a closed-loop control operation, the reference signal and feedback are set to the controller. The experimental platform has native integration with MATLAB/Simulink, which is desirable for conducting tests in a MiL environment. Different control laws can be implemented in numerical simulations for the outer loop to validate the obtained model. This setup allows measurable results in the physical system to directly verify the proposed methodology’s effectiveness.

The next section describes in detail the application of the MbPBO methodology in the RRR robot case study mentioned.

### Stage I: Determination of the base model for the RRR robot and its key parameters

The considered dynamic system is an autonomous system; as such, its physical parameters are invariant in time. This characteristic makes it suitable for creating a parametric model where the parameters can be identified offline [93]. Additionally, the model is also proposed as a non-conservative system. The frictions between links present a challenge objective for the optimal parameterization proposed by the MbPBO methodology because specifying these parameters is difficult for any manufacturer.

A dynamic model of the system is chosen for the primary mathematical modeling effort, where the relationship between the movement and the forces that produce it is explicitly described. For its formulation, it was decided to use the Euler-Lagrange formalism, whose principle is the balance of the kinetic and potential energy of the components of the system. The basis of this model is the Lagrangian equation (13) where $K(q,\dot{q})$ and *P*(*q*) represent, respectively, the kinetic and potential energy of the links.

$$L=\sum_{i=1}^{3}K_{i}(q,\dot{q})-\sum_{i=1}^{3}P_{i}(q)$$ (13)

Likewise, when considering a non-conservative system where energy is dissipated, the Rayleigh dissipation function is included in this formulation. This function models these friction forces as viscous dampers in the term *D* shown in (14).

$$D=\frac{1}{2}\left(\sum_{i=1}^{3}b_{i}{\dot{q_{i}}}^{2}\right)$$ (14)

The term *b*_*i*_ in this last equation represents the damper coefficients modeled for the *i*–*th* link. Including this dissipation function in the model, the corresponding Lagrange equations for the modeling of non-conservative systems are expressed in equation (15).

$$\frac{d}{dt}\left(\frac{\partial L}{\partial \dot{q_{i}}}\right)-\frac{\partial L}{\partial q_{i}}+\frac{\partial D}{\partial \dot{q_{i}}}=0$$ (15)

The model obtained from [94] developed with the Euler-Lagrange energy method was abbreviated in terms to assume the limitations of the simulator and promote shorter calculation times. The latter process considers the third aspect of this first stage related to the model reduction.

The effect of viscous friction for each link is represented as $F_{v}=[f_{v_{1}},f_{v_{2}},f_{v_{3}}]$, and likewise, the vector τ=[τ_1_,τ_2_,τ_4_] represents the input vector of torques.

The closed form of the robot manipulator’s dynamic model is described in (16), where $B\in {\mathbb{R}}^{3\times 3}$ represents the inertia matrix, $C\in {\mathbb{R}}^{3\times 3}$ is the matrix of Coriolis and centrifugal forces, G∈ℝ^3^ is the gravity vector, and $F\in {\mathbb{R}}^{3}$ corresponds to the vector of torques produced by friction.

$$\tau =B(q)\ddot{q}+C(q,\dot{q})\dot{q}+G(q)+F(\dot{q})\in {\mathbb{R}}^{3}$$ (16)

where:


$$B_{11}={Iz}_{1}+{Iz}_{2}+{Iz}_{3}+{l_{1}}^{2}(m_{2}+m_{3})+{l_{2}}^{2}m_{3}+{{lc}_{1}}^{2}m_{1}+{{lc}_{2}}^{2}m_{2}+{{lc}_{3}}^{2}m_{3}$$

$$B_{12}=B_{21}=\epsilon_{2}$$

$$B_{13}=B_{31}=\epsilon_{3}$$

$$B_{22}=m_{3}{l_{2}}^{2}+\epsilon_{4}+m_{2}{{lc}_{2}}^{2}+m_{3}{{lc}_{3}}^{2}+{Iz}_{2}+{Iz}_{3}$$

$$B_{23}=B_{32}=\epsilon_{1}$$

$$B_{33}=I_{z_{3}}+l_{c_{3}}^{2}m_{3}$$ (17)

$$C_{11}=-l_{1}{lc}_{3}m_{3}\delta_{10}(\dot{q_{2}}+\dot{q_{3}})-\delta_{7}-\delta_{5}-\delta_{1}$$

$$C_{12}=-\delta_{8}-\delta_{7}-\delta_{6}-\delta_{5}-\delta_{1}-l_{1}{lc}_{3}m_{3}\delta_{10}\delta_{4}$$

$$C_{13}=-\left(l_{2}{lc}_{3}m_{3}\sin(q_{3})+l_{1}{lc}_{3}m_{3}\delta_{10}\right)\delta_{4}$$

$$C_{21}=\delta_{9}+\delta_{8}+\delta_{6}-\delta_{1}$$

$$C_{22}=-\delta_{1}$$

$$C_{23}=-\delta_{3}-\delta_{2}-\delta_{1}$$

$$C_{31}=\delta_{9}+\delta_{3}+\delta_{2}$$

$$C_{32}=\delta_{3}+\delta_{2}$$ (18)

$$C_{33}=0$$

$$G_{11}=\gamma_{1}+\gamma_{3}+\gamma_{2}+gl_{1}m_{2}\sin(q_{1})+gl_{1}m_{3}\sin(q_{1})+g{lc}_{1}m_{1}\sin(q_{1})$$

$$G_{21}=\gamma_{1}+\gamma_{3}+\gamma_{2}$$

$$G_{31}=\gamma_{1}$$ (19)

$$F_{11}=f_{v_{1}}{\dot{q}}_{1}$$

$$F_{21}=f_{v_{2}}{\dot{q}}_{2}$$

$$F_{31}=f_{v_{3}}{\dot{q}}_{3}$$ (20)

with:


$$\epsilon_{1}=m_{3}{{lc}_{3}}^{2}+l_{2}m_{3}\cos(q_{3}){lc}_{3}+{Iz}_{3}$$

$$\epsilon_{2}=m_{3}{l_{2}}^{2}+\epsilon_{4}+l_{1}m_{3}\cos(q_{2})l_{2}+m_{2}{{lc}_{2}}^{2}+{Iz}_{2}+l_{1}m_{2}\cos(q_{2}){lc}_{2}+m_{3}{{lc}_{3}}^{2}$$

$$+l_{1}m_{3}\epsilon_{5}{lc}_{3}+{Iz}_{3}$$

$$\epsilon_{3}={Iz}_{3}+{{lc}_{3}}^{2}m_{3}+l_{1}{lc}_{3}m_{3}\epsilon_{5}+l_{2}{lc}_{3}m_{3}\cos(q_{3})$$

$$\epsilon_{4}=2m_{3}\cos(q_{3})l_{2}{lc}_{3}$$

$$\epsilon_{5}=\cos(q_{2}+q_{3})$$ (21)

$$\delta_{1}=l_{2}{lc}_{3}m_{3}\sin(q_{3})\dot{q_{3}}$$

$$\delta_{2}=l_{2}{lc}_{3}m_{3}\sin(q_{3})\dot{q_{2}}$$

$$\delta_{3}=l_{2}{lc}_{3}m_{3}\sin(q_{3})\dot{q_{1}}$$

$$\delta_{4}=\dot{q_{1}}+\dot{q_{2}}+\dot{q_{3}}$$

$$\delta_{5}=l_{1}{lc}_{2}m_{2}\sin(q_{2})\dot{q_{2}}$$

$$\delta_{6}=l_{1}{lc}_{2}m_{2}\sin(q_{2})\dot{q_{1}}$$ (22)

$$\delta_{7}=l_{1}l_{2}m_{3}\sin(q_{2})\dot{q_{2}}$$

$$\delta_{8}=l_{1}l_{2}m_{3}\sin(q_{2})\dot{q_{1}}$$

$$\delta_{9}=l_{1}{lc}_{3}m_{3}\delta_{10}\dot{q_{1}}$$

$$\delta_{10}=\sin(q_{2}+q_{3})$$

$$\gamma_{1}=g{lc}_{3}m_{3}\sin(q_{1}+q_{2}+q_{3})$$

$$\gamma_{2}=g{lc}_{2}m_{2}\sin(q_{1}+q_{2})$$

$$\gamma_{3}=gl_{2}m_{3}\sin(q_{1}+q_{2})$$ (23)

The RRR robot dynamics (16) in the state vector $x=[q,\dot{q}]\in {\mathbb{R}}^{6}$ with the control signal $u=\tau \in {\mathbb{R}}^{3}$ is expressed in a simple form as dx/dt=f(x,p,u).

The following is given concerning the steps (i) and (ii) of the Stage I.

#### (i) Selection of the parameters to be estimated

It is then defined for this system (based on Fig 3, and the previously presented model), the vector *p* (24) as the set of parameters to be obtained in the identification of the RRR robot. The vector in (24) includes 12 elements to be estimated, where $L_{c}=[l_{c_{1}},l_{c_{2}},l_{c_{3}}]$ denotes the vector of distances between the axis of movement of each link to its center of mass, $I_{z}=[I_{z_{1}},I_{z_{2}},I_{z_{3}}]$ is the vector of inertia, $M=[m_{1},m_{2},m_{3}]$ is the mass vector, and $F_{v}=[f_{v_{1}},f_{v_{2}},f_{v_{3}}]$ refers to the viscous friction vector. The parameters in *p* are essential for identifying the RRR robot’s dynamic model using the MbPBO methodology.

$$𝐩=[L_{c},I_{z},M,F_{v}]\in {\mathbb{R}}^{12}$$ (24)

#### (ii) Initial values proposed for the parameters

In the process of parameter identification, it is beneficial to begin with prior knowledge of values close to the parameters to be estimated. This approach allows for a constrained search, enhancing the efficiency of model refinement. The initial values for the parameter vector (24), grouped in $\bar{p}$, are sourced from [94]. In that study, the SolidWorks mechanical modeling program was utilized to estimate the parameters of the robot, such as mass, inertia, and mass center of links, as detailed in Table 2.

**Table 2:** Proposed initial dynamic and kinematic parameters $\bar{p}$ for the RRR robot.

Parameter	Description	Value	Units
$l_{c_{1}}$	Mass center of link 1	−0.0704	m
$l_{c_{2}}$	Mass center of link 2	0.09496	m
$l_{c_{3}}$	Mass center of link 3	0.01222	m
$I_{z_{1}}$	Inertia moment of link 1	0.05749530	$kg\;m^{2}$
$I_{z_{2}}$	Inertia moment of link 2	0.00559463	$kg\;m^{2}$
$I_{z_{3}}$	Inertia moment of link 3	0.00106098	$kg\;m^{2}$
*m*_1_	Mass of link 1	6.48912	kg
*m*_2_	Mass of link 2	0.97433	kg
*m*_3_	Mass of link 3	0.38986	kg
$f_{v_{1}}$	Friction coefficient of link 1	0.9	Nms
$f_{v_{2}}$	Friction coefficient of link 2	0.9	Nms
$f_{v_{3}}$	Friction coefficient of link 3	0.9	Nms

In this case, neither the manufacturer nor a CAD program can provide the friction parameter $f_{v_{i}}$. This parameter pertains to the coefficient of friction for dry surfaces, also known as dry friction, which varies depending on the materials in contact. The coefficient of friction between two dry metallic surfaces typically ranges from 0.15 - 0.8 [75]. Therefore, the known range of friction coefficients for metallic surfaces and factors that could result in the highest friction coefficient (like oxidation and high contact pressure, to which the robot joints could be exposed) were considered when proposing the friction coefficient values for the parameter estimation process. In this particular case, one of the worst cases is selected as the initial value for the friction coefficients. So, those values are initially set as 0.9.

### Stage II: Characterization of the RRR robot’s behavior.

**Fig 5:**
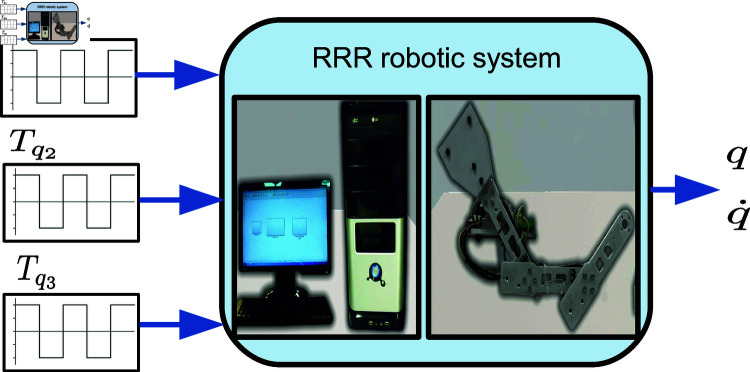
Schematic diagram of the open-loop control experiment conducted on the experimental platform to excite the system states.

To characterize the system behavior, identification experiment on the RRR robot is developed. The experiment used open-loop control signals (open-loop external signal into the outer loop of the cascade system) along with their respective periods, following persistent excitation signals recommended in [95] for this purpose. Each reference signal *ref*_*i*_ to the inner loop controller for each degree of freedom (d.o.f.) has a square wave signal. These signals varied between the maximum applied current $ref_{max}=2.5\;[N\;m]$ that the actuators could handle. Specific periods *T* were interleaved within each reference signal, ensuring an experimentation time $t_{f}=2.6\;[s]$ with a sampling time of 1 [ms]. The experiment induced various behaviors in the robot’s states by applying these signals *ref* in an open-loop control configuration. In this case study, we focused on monitoring the positions and velocities of the robot’s links $\left[q,\dot{q}\right]$. A representative diagram of this experimental setup can be seen in Fig 5.

The individual periods applied to the three degrees of freedom are: $T_{q_{1}}=[0.1,0.2,0.3,$ 0.4,0.1,0.4,0.5,0.6] [s], $T_{q_{2}}=[0.5,0.1,0.4,0.2,0.4,0.1,0.3,0.6]\;[s]$ and $T_{q_{3}}=[0.2,0.3,0.2,$ 0.1,0.3,0.6,0.4,0.5] [s].

**Fig 6:**
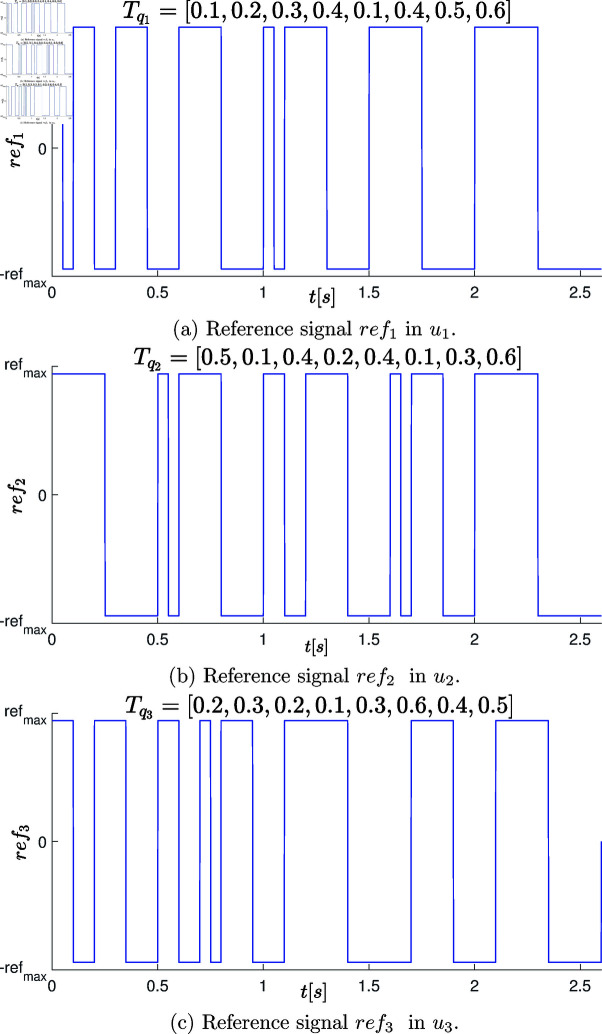
Reference signals used in the open-loop controller on the experimental platform to excite the corresponding system states.

The graphical representation of the reference signals *ref*_1_, *ref*_2_, and *ref*_3_ in the outer loop controller to provide the reference in the inner loop controller of the robot is visualized in Fig 6.

**Fig 7:**
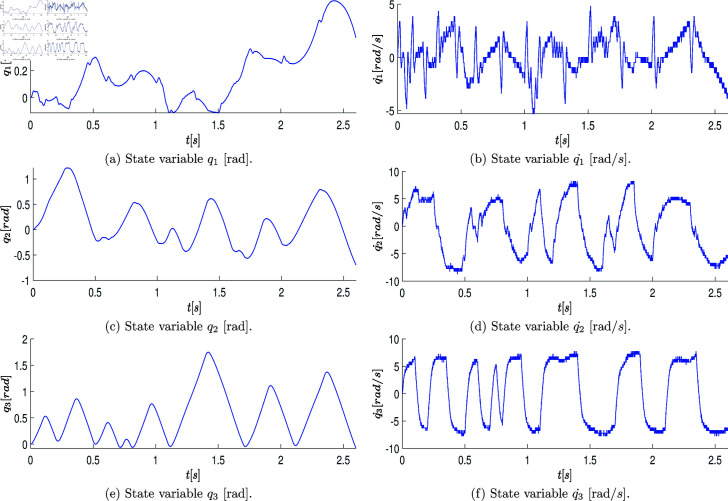
Graphs showing the position state vectors q and the estimated velocity state vectors $\dot{q}$ of the real system when the system states are excited by the open-loop control signals.

At the end of this stage, a data vector of position states defined as $q=[q_{1},q_{2},q_{3}{]}^{T}\in {\mathbb{R}}^{3}$ is obtained, which is directly retrieved from the robot’s encoder over the period of 2.6 [s] (resulting in a state measurement vector of 2600 samples). These measurements are graphically represented with blue lines in the plots of Fig 7a, 7c, 7e. The velocity state, defined as q˙=[q_1_˙,q_2_˙,q_3_˙]_T_∈R_3_, is estimated using numerical differentiation in MATLAB. The plots of Fig 7b, 7d, 7f illustrate the estimated velocity values.

### Stage III: Establishment of a mathematical programming problem for the RRR robot characterization.

With the information from the previous stage, the current reference signal *ref* established in the previous stage is ideally transformed into torque input for only simulation purposes by the equation $\tau_{ref}=k_{m}ref$ [96], where *k*_*m*_ = 1.9839 is the torque constant. So, the control signal $u_{i}=\tau_{ref_{i}}$ is applied in simulation to the degrees of freedom in the robot model defined in Stage I. Its numerical solution provides us with a vector of estimated positions and velocities, defined as $\bar{q}=[\bar{q_{1}},\bar{q_{2}},\bar{q_{3}}{]}^{T}\in {\mathbb{R}}^{3}$ and $\dot{\bar{q}}=[\dot{\bar{q_{1}}},\dot{\bar{q_{2}}},\dot{\bar{q_{3}}}{]}^{T}\in {\mathbb{R}}^{3}$, respectively. The error vector (25) is then defined between the actual position $q\in {\mathbb{R}}^{3}$ and the estimated position $\bar{q}$, as well as, the error vector of the velocity states between the experimental data $\dot{q}\in {\mathbb{R}}^{3}$ and those associated with the model $\dot{\bar{q}}$ (26). Thus, the position and velocity errors of the i-th link are represented by the vectors *e*_*i*_ and ${\dot{e}}_{i}$, respectively.

$$e=q-\bar{q}\in {\mathbb{R}}^{3}$$ (25)

$$\dot{e}=\dot{q}-\dot{\bar{q}}\in {\mathbb{R}}^{3}$$ (26)

So, in the optimization problem, the goal is to minimize the error between the signals obtained from the experiment in Fig 7 and the output of the simulated system in the time domain (error dynamics). Therefore, it is imperative to minimize errors in the states. For this purpose, the criterion of minimizing the integral of the square error (ISE) is chosen, as it is most suitable for this purpose [97]. Once the error vectors have been defined, the objective function terms are described in the space of positions and velocities in (27) and (28), respectively.

$$J_{1}=\int_{0}^{t_{f}}e^{T}e\;dt$$ (27)

$$J_{2}=\int_{0}^{t_{f}}{\dot{e}}^{T}\dot{e}\;dt$$ (28)

Following the indication given in Stage III of the MbPBO methodology description, the multi-objective nature of the problem is transformed into a single-objective problem by implementing a weighted sum approach. Here, dimensionless values $\mu_{1}$ and $\mu_{2}$ represent the weights of the position and velocity errors, respectively. The objective function is minimized based on a normalized weighted expression $\bar{J}$. For this particular case study, the objective function can be defined as in (29), where only two terms are included to provide the same importance for the position errors and other priority for the velocity errors.

$$\bar{J}(p)=\mu_{1}\frac{J_{1}}{\max(J_{1})}+\mu_{2}\frac{J_{2}}{\max(J_{2})}$$ (29)

In (29), the scalar values $\max(J_{1})$ and $\max(J_{2})$ are quantities that represent the maximum possible values of *J*_1_ and *J*_2_ that will produce a dimensionless and normalized value of $\bar{J}$ (ranging between 0 and 1). Those maximum values are obtained by only minimizing one term in the optimization process, i.e., by only minimizing *J*_1_ (27) or *J*_2_ (28), and the obtained optimal design variable vector $p_{J_{1}}^{*}$ or $p_{J_{2}}^{*}$ is evaluated in the other terms. So, in the particular case, the maximum value $\max(J_{2})$ is calculated by evaluating the term *J*_2_ with the obtained vector $p_{J_{1}}^{*}$ (when the *J*_1_ term is minimized), i.e., $\max(J_{2})=J_{2}(p_{J_{1}}^{*})$. In a similar fashion, the maximum value $\max(J_{1})$ is given by minimizing *J*_2_, obtaining the optimal design variable vector $p_{J_{2}}^{*}$ and calculated the maximum value $\max(J_{1})$ as $\max(J_{1})=J_{1}(p_{J_{2}}^{*})$. The specific values of $\max(J_{1})$ and $\max(J_{2})$ are provided in the next Stage IV.

The dynamic constraints (35) related to the system behavior are included in the optimization problem to bind the problem solution to the dynamic behavior of the RRR robot (16).

A tolerance range is established around the initial (base) value $\bar{p}$ of each variable *p*_*i*_ to limit the search space in the optimization process. The increment or reduction factor is denoted as $\Delta {\bar{p}}_{i}={\bar{p}}_{i}\frac{p_{v}}{100}$, where $p_{v}$ represents the desired percentage change over the base value $\bar{p}$ shown in Table 2. The chosen increment/reduction factor values impose practical boundaries in the design variables to ensure that the search space in the optimization process presents valid solutions. The optimization process with these bounds is also more efficient because it does not waste time looking for solutions where the system can not operate. An increase and decrease factor of at most thirty percent is recommended for this purpose. Equations (30) to (33) describe such a limits ($p_{min_{i}}\leq p_{i}\leq p_{max_{i}},\;\forall \;\;i=[1,...,n]$) and define how the bound constraints are given. The selected upper and lower bound ranges (${\bar{p}}_{i}$ ± $\Delta {\bar{p}}_{i}$) are obtained from their initial (base) values $\bar{p}$ and the increment/reduction factor $\Delta \bar{p}$ given in Table 3. So, the bounds of the design variable vector (30)-(33) are the other constraints taken into account in the optimization problem. In (29), the scalar values $\max(J_{1})$ and $\max(J_{2})$ are quantities that represent the maximum possible values of *J*_1_ and *J*_2_ that will produce a dimensionless and normalized value of $\bar{J}$ (ranging between 0 and 1). Those maximum values are obtained by only minimizing one term in the optimization process, i.e., by only minimizing *J*_1_ (27) or *J*_2_ (28), and the obtained optimal design variable vector $p_{J_{1}}^{*}$ or $p_{J_{2}}^{*}$ is evaluated in the other terms. So, in the particular case, the maximum value $\max(J_{2})$ is calculated by evaluating the obtained vector $p_{J_{1}}^{*}$ when the *J*_1_ term is minimized, i.e., $\max(J_{2})=J_{2}(p_{J_{1}}^{*})$. In a similar fashion, the maximum value $\max(J_{1})$ is given by minimizing *J*_2_, obtaining the optimal design variable vector $p_{J_{2}}^{*}$ and with this information can compute the maximum value $\max(J_{1})$ as $\max(J_{1})=J_{1}(p_{J_{2}}^{*})$. The specific values of $\max(J_{1})$ and $\max(J_{2})$ are provided in the next Stage IV.

**Table 3:** Percentage rate $p_{v}$ in the design variable changes and the design variable bounds used in the optimization process. The specific quantities are indicated with $\Delta {\bar{p}}_{i}$.

Parameter ${\bar{p}}_{i}$	$p_{v}$ (Tolerance rate)	$\Delta {\bar{p}}_{i}$	$p_{min_{i}}$	$p_{max_{i}}$
${\bar{p}}_{1}$	25%	0.017620	-0.0880200	-0.052780
${\bar{p}}_{2}$	25%	0.023740	0.07122000	0.118700
${\bar{p}}_{3}$	25%	0.003055	0.00916500	0.0152750
${\bar{p}}_{4}$	25%	0.014373	0.04312200	0.0718680
${\bar{p}}_{5}$	25%	0.001400	0.00419460	0.0069946
${\bar{p}}_{6}$	25%	0.000275	0.00078598	0.0013360
${\bar{p}}_{7}$	10%	0.648912	5.84020000	7.1380000
${\bar{p}}_{8}$	10%	0.097433	0.87690000	1.0718000
p¯9	10%	0.038986	0.35087000	0.4288500
${\bar{p}}_{10}$	30%	0.270	0.63000000	1.1700000
${\bar{p}}_{11}$	25%	0.225	0.67500000	1.1250000
${\bar{p}}_{12}$	25%	0.225	0.67500000	1.1250000

The dynamic constraints (35) related to the system behavior are included in the optimization problem to bind the problem solution to the dynamic behavior of the RRR robot (16).

A tolerance range is established around the initial (base) value ($\bar{p}$) of each variable *p*_*i*_ to limit the search space in the optimization process. The increment or reduction factor is denoted as $\Delta {\bar{p}}_{i}={\bar{p}}_{i}\frac{p_{v}}{100}$, where $p_{v}$ represents the desired percentage change over the base value $\bar{p}$ shown in Table 2. The chosen increment/reduction factor values impose practical boundaries in the design variables to ensure that the search space in the optimization process presents valid solutions. The optimization process with these bounds is also more efficient because it does not waste time looking for solutions where the system can not operate. An increase and decrease factor of at most thirty percent is recommended for this purpose. Equations (30) to (33) describe such a limits ($p_{min_{i}}\leq p_{i}\leq p_{max_{i}},\;\forall \;\;i=[1,...,n]$) and define how the bound constraints are given. The selected upper and lower bound ranges (${\bar{p}}_{i}\pm \Delta {\bar{p}}_{i}$) are obtained from their initial (base) values $\bar{p}$ given in Table 2 and the increment/reduction factor $\Delta \bar{p}$ given in Table 3. So, the bounds of the design variable vector (30)-(33) are the other constraints taken into account in the optimization problem.

$$\overset{p_{min_{i}}}{\overbrace{l_{c_{i}}\;-\;\Delta {\bar{p}}_{i}}}\leq \;\;p_{i}\;\leq \overset{p_{max_{i}}}{\overbrace{l_{c_{i}}\;+\;\Delta {\bar{p}}_{i}}}$$ (30)

$$I_{z_{i}}-\Delta {\bar{p}}_{i+3}\leq p_{i+3}\leq I_{z_{i}}+\Delta {\bar{p}}_{i+3}$$ (31)

$$m_{i}-\Delta {\bar{p}}_{i+6}\leq p_{i+6}\leq m_{i}+\Delta {\bar{p}}_{i+6}$$ (32)

$$f_{v_{i}}-\Delta {\bar{p}}_{i+9}\leq p_{i+9}\leq f_{v_{i}}+\Delta {\bar{p}}_{i+9}$$ (33)

Relating the above with the formal approach, the problem consists of finding the vector of design variables *p* that minimizes the weighted objective function $\bar{J}$ (34), subject to the dynamic behavior of the system model (35), and the constraints on the design variables (36). In this case, the equality constraints are implicitly satisfied by the solution of the model dynamics.

$$\underset{p\in \Omega^{n}}{\text{Min}}\;\bar{J}(p)$$ (34)

subject to:

$$\frac{d\bar{x}}{dt}=f(\bar{x},p,u)$$ (35)

$$p_{min,i}\leq p_{i}\leq p_{max,i},\forall \;i=[1,...,n]$$ (36)

The technique applied to obtain the minimum ${\bar{J}}^{*}$ among these errors will yield the vector of optimal design variables p^*^, where $p\in {\mathbb{R}}^{n}$ and *n* = 12 (24). The optimization techniques used to solve this problem will be addressed in the following Stage IV.

### Stage IV: Solving the RRR robot’s characterization problem using bio-inspired algorithms

It is important to point out that both terms of the objective function (29) present trade-offs for fulfilling both criteria. This means that minimizing both errors is not possible, i.e., the relationship between them turns out to be inverse; they behave like two opposing objectives where only minimizing the first term *J*_1_ implies maximizing the second one *J*_2_ and vice versa.

In order to limit the weight values $\mu_{1}$ and $\mu_{2}$ to $\mu_{1}+\mu_{2}=1$, it is important to normalize the terms (to set the values of such terms in the interval between [0,1]) of the objective functions. So, the terms max(J_1_) and max(J_2_) of (29) are obtained by individually minimizing the terms *J*_2_ and *J*_1_, respectively, and using the same constraints in (35)-(36), as described in the previous stage. In this part of the methodology, it is recommended to set several independent runs (for instance, thirty times [89]) for solving both optimization problems due to the stochastic behavior of the algorithm. DE/rand/1/bin is used in this case, but other frequently used bio-inspired optimizers can also be used. The best execution, the one that has the minimum value of the term, is selected from the thirty runs. The result that further minimizes the term *J*_1_ provides the objective function vector $J(p_{J_{1}}^{*})=[J_{1},\;J_{2}]=[0.080279204,\;31.775743289]$. Meanwhile, the result that further minimizes the term *J*_2_ provides the objective function vector $J(p_{J_{2}}^{*})=[0.196757289,\;29.034641825]$. The values in boldface are the possible extremes sought, i.e., $\max(J_{1})=0.196757289$ and $\max(J_{2})=31.775743289$. For practical simplicity, the values that are considered to carry out the normalization in the equation (29) are J1MAX=max(J1)=0.2 and J2MAX=max(J2)=32. Once the maximum values of the terms in the optimization problem are obtained, the three steps considered in this stage are described below.

#### (i) Trade-off selection

As mentioned in the description of the MbPBO methodology, the multi-objective problem is transformed into a single-objective problem by a weighted sum approach. A set of non-dominated solutions must be generated by establishing different weights in the optimization problem (34)-(36), forming the approximate Pareto front in the performance function space. The Pareto front helps the designer make decisions on the most suitable trade-off for the specific application.

Once the terms of the objective function $\bar{J}$ are normalized, new experiments are carried out to find the best trade-offs in the terms *J*_1_ and *J*_2_ (quadratic errors of position and velocity). Then, fourteen combinations of weights $\mu_{1}$ and $\mu_{2}$ in the interval [0,1] are tested. The first two combinations find the solution of the Pareto front without a trade-off, i.e., by setting $\mu =[\mu_{1},\mu_{2}]=[1,0]$ and $\mu =[\mu_{1},\mu_{2}]=[0,1]$. The next nine combinations consider uniform intervals from μ=[0.9,0.1] to μ=[0.1,0.9] subject to $\mu_{1}$  +  $\mu_{2}=1$. The three final weights are assigned according to the best promising weight interval found in the previous experiment. The promising weight interval is visualized once the previous experiments with different weights are carried out. Thirty independent executions of the bio-inspired algorithm are performed by establishing different weights in the optimization problem (34)-(36) and using the same DE algorithm with the same parameters given above. This experiment aims to identify the combination of weights that results in a helpful trade-off solution by including the best solution (the solution that further minimizes the performance function from the algorithm executions) found in each combination.

**Fig 8:**
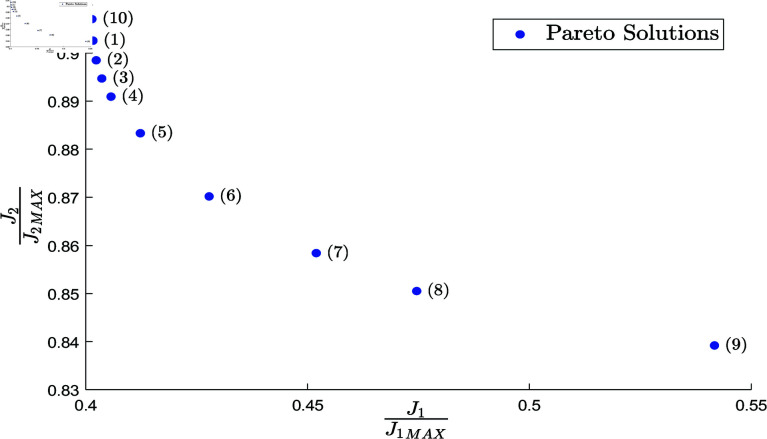
Normalized Pareto front obtained by using different weights in the optimization process.

Table 4 shows the chosen weight values, the weighted normalized objective function $\bar{J}$, and its weighted non-normalized terms *J*_1_ and *J*_2_. The values in boldface indicate the minimum ones in each column. As the uniform distribution of weights does not guarantee uniform distribution of the found Pareto solutions [98], and even the bio-inspired optimizer can stagnate in local solutions [83], the obtained solutions are sorted according to non-dominance verification. The Pareto solutions [83] of results from Table 4 are displayed in Table 5 and the graphical representation of the Pareto front with the normalized terms ${\bar{J}}_{1}$ and ${\bar{J}}_{2}$ is shown in Fig 8.

**Table 4:** Values of the objective function $\bar{J}$ and its non-normalized terms $J_{1}$, $J_{2}$ obtained by using different weights in the optimization process.

$\mu_{1}$	$\mu_{2}$	$\bar{J}$	$J_{1}$	$J_{2}$
1	0	**0.401396023**	0.080279204	31.775743289
0.995	0.005	0.403922310	**0.080278789**	31.747354780
0.999	0.001	0.401903781	0.080279576	31.755360737
0.99	0.01	0.406465435	0.080281565	31.750901927
0.9	0.1	0.451735104	0.080328387	31.590077207
0.8	0.2	0.501576125	0.080469485	31.447181929
0.7	0.3	0.550949727	0.080726310	31.314224737
0.6	0.4	0.599784629	0.081139216	31.182110801
0.5	0.5	0.647818780	0.082462203	30.916428990
0.4	0.6	0.693224894	0.085562030	30.455881984
0.3	0.7	0.736482439	0.090392267	30.044701936
0.2	0.8	0.775334616	0.094920532	29.768116156
0.1	0.9	0.809449550	0.108345124	29.371882860
0	1	0.829561195	0.196757289	**29.034641825**

**Table 5:** Set of Pareto solutions obtained by the results of Table 4.

NDS	$\mu_{1}$	$\mu_{2}$	$\bar{J}$	$J_{1}$	$J_{2}$
1	0.9	0.1	0.45173	0.08032	31.59007
2	0.8	0.2	0.50157	0.08046	31.44718
3	0.7	0.3	0.55094	0.08072	31.31422
4	0.6	0.4	0.59978	0.08113	31.18211
5	0.5	0.5	0.64781	0.08246	30.91642
6	0.4	0.6	0.69322	0.08556	30.45588
7	0.3	0.7	0.73648	0.09039	30.04470
8	0.2	0.8	0.77533	0.09492	29.76811
9	0.1	0.9	0.80944	0.10834	29.37188
10	0.995	0.005	0.40392	0.08027	31.74735

Once the Pareto front is obtained, the decision maker’s preference provides one useful trade-off for the application. In this case, the most preferred solution is the Pareto solution with the minimum value in the term *J*_1_ and with a suitable value in the term *J*_2_. Based on Table 5 and Fig 8, the combination of weights $\mu_{1}=0.995$, $\mu_{2}=0.005$ stands out for its ability to further minimize the trade-off in the performance function $\bar{J}$ and the position quadratic errors, also providing an acceptable velocity quadratic error. The selected weights are employed in the next step to analyze different bio-inspired algorithms to optimize the RRR robot’s characterization problem.

#### (ii) Algorithm selection

The choice of the DE/rand/1/bin algorithm for the experiments performed previously is because it is one of the most widely used alternatives in the reviewed literature [99] (Fig 1). However, the MbPBO methodology description in this work suggests using two more techniques to solve the optimization problem with the selected obtained weights ($\mu_{1}=0.995$, $\mu_{2}=0.005$). Therefore, the next frequently used algorithms in the reviewed literature are selected due to their adaptability, population-based strategy, robust performance, and simplicity [85–87]. In particular, the Particle Swarm Optimization (PSO) [39] with the best global topology and the Genetic Algorithm (GA) in its generational variant [100] are also selected to optimize the RRR robot’s characterization problem described before (34)-(36).

As mentioned in the methodology description, it is only possible to determine which algorithm presents the best results for a specific problem once it is experimentally proven [88]. Therefore, given the previous algorithms, the present work proposes a comparison based on the results obtained to identify which bio-inspired technique generally performs best when solving the characterization problem. The algorithm parameters must be tuned either “by hand” through systematic manual settings, “by analogy” through the information suggested in the state-of-the-art for similar problems, or using self-tuning mechanisms. The process “by hand” (where algorithm parameters are varied sequentially and ordered to select the best configuration that solves the problem) is also suggested in [101] for algorithms like DE. Therefore, the latter is the process chosen in this work to tune such parameters. The set of parameters presented below was obtained from this trial and error procedure, where the final tuning values for the DE/rand/1/bin algorithm are as follows: Maximum number of generations *G*_*max*_ = 500, number of individuals *NP* = 100, scaling factor F∈[0.1,0.9], and crossover rate *CR* = 0.2. In the case of PSO, the following values are obtained: Maximum number of generations *G*_*max*_ = 500, number of individuals *NP* = 100, weight of the current best *C*_1_ = 1.8, weight of the global best *C*_2_ = 2.2 and speed factor ω=0.729. The parameters for the GA are set as: Maximum number of generations *G*_*max*_ = 500, number of individuals *NP* = 100, mutation probability *PM* = 1/6, and crossover probability *PC* = 0.9. It is important to point out that the computational complexity of PSO and GA is similar to DE [102]. This is obtained by the number of calls to the objective function evaluation $c(\bar{J})$, the maximum iteration number *G*_*max*_, and the population size *NP*. Then, the computational complexity in a “big O” notation is given by $O(c(\bar{J})$ · *NP* · *G*_*max*_). So, in all algorithms, the total evaluation number of the objective function is 50000 to make a fair comparison in the next step.

#### (iii) Performance analysis of bio-inspired algorithms

Thirty independent runs were performed for each of the above algorithms. Each algorithm was programmed in the M language of MATLAB^TM^, on a commercial PC with an Intel^TM^ Core i7 @ 3.2 GHz processor, 16 GB of RAM. The descriptive statistics are presented below based on the criteria of the normalized weighted error $\bar{J}$. The average (*mean*), standard deviation σ, the best (minimum), and the worst (maximum) are specified based on the data obtained from the best individual of the last generation of each run. Tables 6-8 summarize the results obtained by each algorithm for the different criteria.

**Table 6:** Summary of descriptive statistics in $\bar{J}$ for the optimization process of thirty executions with different bioinspired algorithms.

Criteria	DE/Rand/1/bin	PSO	GA
best($\bar{J}$)	0.403939125	0.403900924	0.403900930
worst($\bar{J}$)	0.404164242	0.409905613	0.404171660
$\sigma (\bar{J})$	$4.64\times 10^{-5}$	0.002411968	$5.68\times 10^{-5}$
mean($\bar{J}$)	0.404005969	0.405259492	0.403928309

**Table 7:** Summary of descriptive statistics in $J_{1}$ for the optimization process of thirty executions with different bioinspired algorithms.

Criteria	DE/Rand/1/bin	PSO	GA
best(*J*_1_)	0.080282092	0.080274268	0.080274308
worst(*J*_1_)	0.080325148	0.081414481	0.080326348
$\sigma (J_{1})$	$8.89\times 10^{-6}$	$4.57\times 10^{-4}$	$1.08\times 10^{-5}$
mean(*J*_1_)	0.080294948	0.080532313	0.080279657

**Fig 9:**
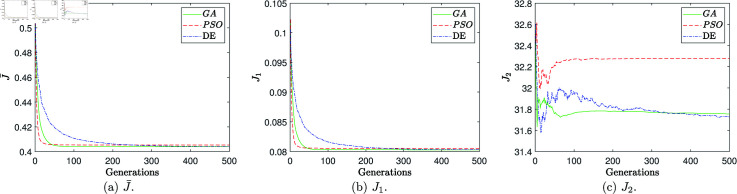
Mean convergence graphs of the evolution in the performance function and its terms through generations in thirty executions of each algorithm.

The tables show that the PSO algorithm gets the best solution in terms of the objective function $\bar{J}$, as seen in Table 6. This is highlighted in boldface. Also, PSO presents the same behavior behavior concerning the individual objective function terms *J*_1_ and *J*_2_ in Tables 7 and 8. The solutions obtained with the DE techniques and GA for $\bar{J}$ turn out to be very close to PSO with a percentage increment of 0.0094% for DE and 0.00000148% for GA. Additionally, the plots in Fig 9a shows a follow-up of the evolution through generations of the mean objective function of the best solutions in thirty executions (convergence graph of the mean objective function) using different optimization techniques. It is observed that while PSO converges the fastest, its overall performance through the algorithm executions is not as good on average. For completeness, Fig 9b and 9c also shows the convergence graph of the mean objective function terms using different optimization techniques, i.e., the evolution of the position error term *J*_1_ and the velocity error term *J*_2_ through generations are displayed. It is observed that the velocity error term *J*_2_ presents more oscillations before the three hundred generations than the term *J*_1_, where the *J*_1_ behavior is more stable from one hundred generations. This indicates that the achievement of minimizing the position error term *J*_1_ is more evident than the velocity error term *J*_2_.

**Table 8:** Summary of descriptive statistics in $J_{2}$ for the optimization process of thirty executions with different bioinspired algorithms.

Criteria	DE/Rand/1/bin	PSO	GA
best(*J*_2_)	30.733128258	30.336899969	30.446928955
worst(*J*_2_)	31.652124398	32.419766014	31.732273887
$\sigma (J_{2})$	0.228855752	0.509185588	0.313453826
mean(*J*_2_)	31.204262966	31.283389837	31.251702794

**Table 9:** Design variable vector $p^{*}$ obtained with different bioinspired techniques.

Parameter	DE/Rand/1/bin	PSO	GA
$l_{c_{1}}$	−0.0678173	−0.0681964	−0.0681981
$l_{c_{2}}$	0.11868310	0.11870000	0.11869999
$l_{c_{3}}$	0.01041319	0.01047347	0.01047617
$I_{z_{1}}$	0.07185556	0.07186912	0.07186912
$I_{z_{2}}$	0.00699969	0.00700000	0.00700000
$I_{z_{3}}$	0.00137492	0.00137500	0.00137499
*m*_1_	5.86299858	5.84020895	5.84020896
*m*_2_	0.87691113	0.87689700	0.87689700
*m*_3_	0.35088631	0.35087400	0.35087400
$f_{v_{1}}$	1.16969739	1.16999999	1.16999999
$f_{v_{2}}$	0.68373018	0.68363457	0.68364980
$f_{v_{3}}$	0.80259328	0.80217106	0.80216750

**Fig 10:**
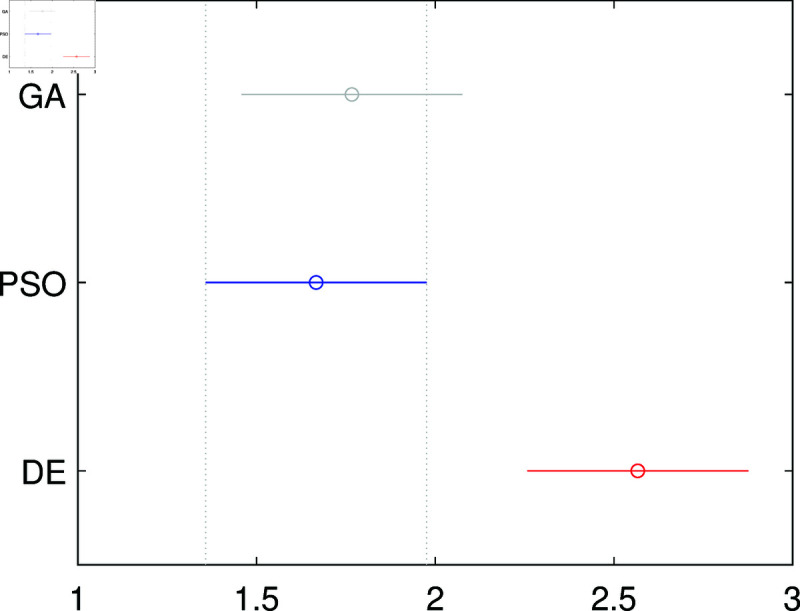
The Bonferroni multiple comparison post hoc test with a significance level of α = 0.05. The control method is PSO.

Even though in Fig 9a the evolution in PSO of the mean objective function shows the worse performance through generations, PSO obtained a variety of solutions that resulted in an average $mean(\bar{J})$ and standard deviation $\sigma (\bar{J})$ higher than those of the other two algorithms (see Tables 6–8). PSO also demonstrates a greater search space exploration (see standard deviation), given these sparse solutions, in contrast to the other two algorithms. Despite this, all algorithms can find solutions with comparable performance. In general, the three algorithms, convergence to a value in the case of the weighted target $\bar{J}$ and the term *J*_1_ occurs relatively quickly after two hundred generations. In contrast, the term *J*_2_ manifests a more irregular behavior until the end of the generations. In order to know about the algorithm performance and to generalize the results, the inferential statistics [89] is applied to confirm the performance among algorithms. In particular, the Friedman test followed by Bonferroni post hoc multiple comparisons with a significance level α=0.05 is used. Fig 10 shows the multiple comparison test considering PSO as the control method, where the winner is the algorithm that ranks closest to the left (x-axis in the illustration). Overlapping gray lines show no discernible difference between the algorithms, meaning that no conclusions about the data distributions can be drawn because the p-values in those comparisons are greater than the selected significance level. The red line that does not overlap indicates that the p-value is below the selected significance level in algorithm outcomes, meaning that the results differ significantly. The red line shows that PSO is similar to GA, indicating that both are the most promising algorithms for this problem. PSO and GA also outperform the DE behavior.

On the other hand, Table 9 presents the vectors of resulting design variables p^*^ corresponding to the best solution concerning the performance function $\bar{J}$ of the thirty runs reported in Table 6 for all algorithms. Table 10 compares the obtained parameters previously reported in Table 2 with those obtained using different algorithms, evidencing a certain percentage of variation among them. In the case of the center of mass $l_{c_{i}}$, the variation ranges from 3.2429% to 25%; the inertia $I_{z_{i}}\;\forall \;i=1,2,3$ ranges from 24.9764% to 25%; the mass *m*_*i*_ varies between the interval [9.6488,10]%; and in the case of the friction $f_{v_{i}}$, the variation interval is [10.8230,30]%. The obtained parameters are remarkably similar across the three algorithms, and those algorithms are suitable for solving the problem. However, the parameters obtained by the PSO are used because it achieves the best performance in minimizing the objective function (albeit by a small margin).

**Table 10:** Percentage values of variation of the parameters reported in Table 2 with those obtained using different algorithms in the RRR robot.

Parameter	Base	% Var.	% Var.	% Var.
	value	DE	PSO	GA
$l_{c_{1}}$	−0.0704	3.78	3.24	3.24
$l_{c_{2}}$	0.09496	24.98	25	24.99
$l_{c_{3}}$	0.01222	14.78	14.29	14.27
$I_{z_{1}}$	0.05749530	24.97	25	25
$I_{z_{2}}$	0.00559463	24.99	25	25
$I_{z_{3}}$	0.00106098	24.99	25	24.99
*m*_1_	6.48912	9.64	10	10
*m*_2_	0.97433	9.99	10	10
*m*_3_	0.38986	9.99	9.99	10
$f_{v_{1}}$	0.9	29.96	30	29.99
$f_{v_{2}}$	0.9	24.03	24.04	24.038
f_v3_	0.9	10.82	10.86	10.87

On the other hand, the values of the objective function terms with the obtained parameters by the MbPBO methodology using PSO and with the initial parameters in Table 2 are displayed in Table 11. It is observed that the parameters obtained by the proposal, the terms associated with the position error *J*_1_ and the velocity error *J*_2_ improve their performances in 85.07% and 9.93%, respectively, regarding the obtained performance by the initial parameters. So, significant differences in the objective function performance result from the parameters obtained by the proposed MbPBO methodology, which aids in better characterizing the dynamic model in the MiL simulation.

**Table 11:** Results in the performance of the objective function terms obtained by the MbPBO methodology using PSO with respect to the initial parameters in Table 2.

Design parameters	$J_{1}$	$J_{2}$
Proposal with PSO	0.08027	31.74735
Initial parameters	0.53795	35.24912

#### Comparative analysis with direct search methods

This section analyzes the performance of direct search algorithms in solving the optimization problem related to the robot’s characterization under the proposed MbPBO methodology. The primary goal is to evaluate the trade-offs involved in using other heuristic-based optimizers with respect to bio-inspired optimization.

The selected direct search optimizers include Constrained Optimization BY Linear Approximations (COBYLA), Nelder Mead, and Powell algorithms [103]. Each algorithm is executed independently thirty times using randomly generated initial conditions. The stop criterion is based on the number of objective function evaluations. The objective function evaluation number is 50000, which is related to the same value used in the bio-inspired algorithms.

The descriptive statistic is presented in Table 12. The table includes the average (*mean*), standard deviation (σ), and the minimum (best) and the maximum (worst) performance function values obtained from executions. Additionally, the number of times each algorithm finds the minimum values is presented in parentheses next to the corresponding result.

**Table 12:** Summary of descriptive statistic in $\bar{J}$ for the optimization process of thirty executions with direct search methods.

Criteria	COBYLA	Nelder Mead	Powell
best($\bar{J}$)	0.426154 (1)	0.403917 (1)	0.406864 (1)
worst(J¯)	1.215647	0.411551	0.426008
σ(J¯)	0.264457	0.002930	0.004640
mean(J¯)	0.847217	0.406708	0.414684

Based on the results presented in Table 12, the most promising direct search optimizer is Nelder–Mead, as it achieves the lowest performance function value of $\bar{J}=0.403917$. Powell’s method takes second place with $\bar{J}=0.406864$, followed by COBYLA, which yields a $\bar{J}\;=\;0.426154$. Notably, each algorithm reaches its minimum value only once out of thirty independent executions, leading to relatively high standard deviation values and indicating a lack of consistency across runs.

The comparison between bio-inspired algorithms and direct search methods (Tables 6 and 12) reveals that bio-inspired techniques not only yield superior performance values but also demonstrate higher robustness and repeatability across multiple runs.

#### Comparative analysis with indirect search methods

Similar to the previous section, this section analyzes the performance of indirect search methods (gradient-based algorithms) in solving the optimization problem related to the robot’s characterization. The main objective is to show the importance of bio-inspired optimization in the proposed MbPBO methodology.

The selected gradient-based optimizers include Sequential Least Squares Quadratic Programming (SLSQP), the Limited-memory Broyden–Fletcher–Goldfarb–Shanno with Box constraints (L-BFGS-B), the Truncated Newton Conjugate-Gradient (TNC) and Trust-Region (T-R) algorithms [104]. The same experimental setup, namely, thirty independent runs with randomly initialized conditions and a stopping criterion (50000 objective function evaluations), is adopted as in the previous analysis.

The average (*mean*), standard deviation (σ), minimum (best), and maximum (worst) performance function values obtained from the algorithm executions are presented in Table 13. The frequency at which each algorithm achieved the minimum value is shown in parentheses.

**Table 13:** Summary of descriptive statistic in $\bar{J}$ for the optimization process of thirty executions with indirect search methods (gradient-based algorithms).

Criteria	SLSQP	L-BFGS-B	TNC	T-R
best($\bar{J}$)	0.403901 (17)	0.403901 (10)	0.407030 (1)	0.403902 (2)
worst($\bar{J}$)	0.404092	0.431830	0.539321	2.148789
$\sigma (\bar{J})$	0.000047	0.005728	0.033438	0.434898
mean($\bar{J}$)	0.403926	0.406376	0.431607	0.859620

The most promising gradient-based optimizers are SLSQP and L-BFGS-B based on the results presented in Table 13 because both find solutions with the performance function value of $\bar{J}=0.403901$. Nevertheless, SLSQP stands out by reaching this optimal value seventeen times, indicating greater consistency. The Trust-Region (T-R) takes second place but only finds two times the performance function value $\bar{J}=0.403902$. The worst gradient-based optimizer is TNC because it exhibits the largest performance function value and only finds the sub-optimal solution once out of thirty executions.

It is also observed that the most promising gradient-based optimizers (SLSQP and L-BFGS-B) locate the best solution obtained with the most promising bio-inspired algorithms (PSO and GA in Table 6). Nevertheless, the gradient-based algorithms are very sensitive to the initial conditions, providing a diverse set of local solutions, as described in the standard deviation of Table 13.

Although certain gradient-based algorithms (such as SLSQP and L-BFGS-B) exhibit moderate consistency achieving the optimal solution in around half of thirty executions, the advantage of bio-inspired algorithms (see Tables 6 and 13) lies in their global search capabilities and robustness to problem characteristics such as non-smoothness noise, and complex constraint landscapes. While gradient-based methods may converge efficiently when the search space is well-behaved (e.g., smooth and convex), they depend highly on initial conditions. They may also become trapped in local minima in more irregular or multimodal functions. On the other hand, bio-inspired algorithms incorporate exploration mechanisms to avoid local optima and search for in a wider design space region. These advantages are observed in the lower standard deviation of Table 6, indicating greater consistency in the obtained results. Thus, even if a subset of gradient-based methods performs similarly on this specific problem, bio-inspired algorithms present a more versatile and reliable option across diverse optimization problems where gradient information, constraint inclusion, multi-objective formulations, or parallel implementation is challenging. This makes bio-inspired algorithms particularly well-suited for the MbPBO methodology, which benefits from robust global search strategies and adaptability to complex problem landscapes.

With these findings, the MbPBO methodology can be extended to using hybrid techniques known as memetic algorithms. The memetic algorithm combines the global exploration capabilities of the bio-inspired algorithms with the fine search (exploitation capabilities) of gradient-based techniques. This hybrid extension represents a possible future work in the proposed methodology.

### Stage V: Validation of the obtained RRR robot model and its use in the MiL simulation

Once the comparison described in the step iii) of Stage IV has been carried out, and the design variable vector resulting in the minimum value of the weighted objective function (p^*^ obtained with PSO reported in Table 6) has been obtained, the last stage of the proposed MbPBO methodology is the comparative evaluation concerning the similarity of the system model’s states with those of the plant. So, the first step analyzes the model obtained in open-loop control with the reference signal from Stage II.

In addition, this stage also focuses on assessing the parameterized model’s ability to accurately replicate the behavior of the testbed platform in a wide range of applications to test different MiL simulation environments. This evaluation essentially tests the model’s predictive capability with different control strategies and monitoring scenarios by comparing position and velocity states between the model and the actual testbed platform during specific tasks. So, the second step of this stage analyses the model obtained in closed-loop for the regulation and tracking tasks.

In this stage, graphic comparisons and the Pearson correlation coefficient analysis between experimental and simulation signals are performed to assess the similarity or variability between the system model’s states and the plant’s numerically. The steps of this stage are described below.

#### (i) Model validation in open-loop control

**Fig 11:**
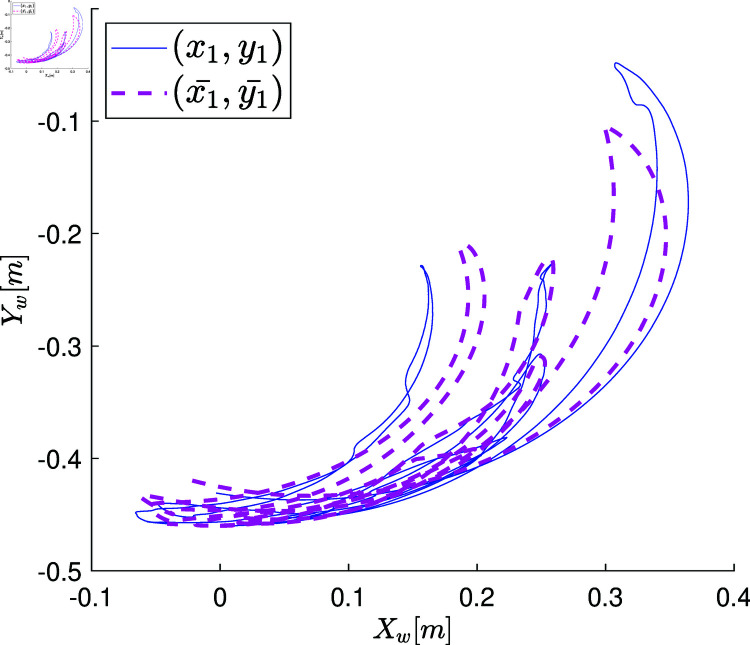
Robot’s end-effector behavior in the Cartesian plane $\left[X_{w},Y_{w}\right]$ with the solution $p^{*}$ obtained by PSO in simulation, against the real states of the experiment when system states are excited by the open-loop control signals.

**Fig 12:**
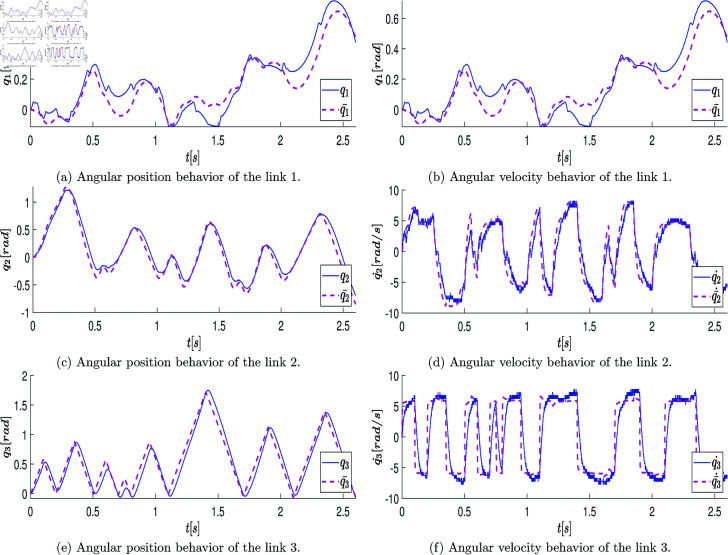
Angular position and velocity behaviors of the RRR robot when the system states are excited by the open-loop control signals. Real experiment (*q*_1_, *q*_2_, *q*_3_, $\dot{q_{1}}$, $\dot{q_{2}}$, $\dot{q_{3}}$) vs. approximated model ($\bar{q_{1}}$, $\bar{q_{2}}$, $\bar{q_{3}}$, $\dot{\bar{q_{1}}}$, $\dot{\bar{q_{2}}}$, $\dot{\bar{q_{3}}}$) with the solution p^*^ obtained by PSO.

Figs 11 and 12 show the Cartesian angular position and angular velocity behavior of the RRR robot obtained from the approximated model (signal displayed in solid blue line) and the real experiment (signal represented by the dotted magenta line). The Cartesian position in the real experiment and the simulated plant is represented by $x_{1},y_{1}$ and $\bar{x_{1}},\bar{y_{1}}$, respectively. Similarly, the angular positions are defined by *q*_1_, *q*_2_, *q*_3_ for the real experiment and $\bar{q_{1}}$, $\bar{q_{2}}$, $\bar{q_{3}}$ for the behavior of the obtained model. The angular velocities are set as $\dot{q_{1}}$, $\dot{q_{2}}$, $\dot{q_{3}}$ for the real experiment and $\dot{\bar{q_{1}}}$, $\dot{\bar{q_{2}}}$, $\dot{\bar{q_{3}}}$ for the simulated plant.

For a better numerical appreciation of the results observed in Figs 11 and 12, Table 14 presents the correlation coefficient ρ between the real measurements and the results of the approximated model for each state previously examined. The coefficient ρ is a statistical measure that indicates the strength and direction of the linear relationship between two variables (Pearson correlation). So, this measure would indicate the similarity (linear relationship) between the data obtained by the model and the experimental data. A coefficient of -1 would indicate a perfect inverse correlation, i.e., with the same input data, the model provides an output with the same magnitude but in an opposite direction than the output of the real system. The value of 0 indicates no correlation, which means that the model data and the experimental data do not exhibit a linear relationship. Hence, the model could not be helpful to describe the experimental (real) behavior. The value of 1 indicates a perfect positive correlation (strong correlation) between the variables, i.e., the model data matches perfectly to the experimental data with the same input data, meaning that the model is useful for describing the real behavior.

**Table 14:** Correlation coefficient ρ between the robot signals and the approximated model behavior when the system states are excited by the open-loop control signals.

State	ρ
vector	($\mu_{1}=0.995,\mu_{2}=0.005$)
*q*_1_	0.946306694
*q*_2_	0.985573578
*q*_3_	0.964797035
$\dot{q_{1}}$	0.672351812
$\dot{q_{2}}$	0.965956984
$\dot{q_{3}}$	0.874157909
Cartesian workspace in *X*_*w*_	0.963192532
Cartesian workspace in *Y*_*w*_	0.830539196

The results show suitable relationships between the actual behavior and the approximated model with a average correlation of 0.9015 in the angular space and 0.8969 in the Cartesian space. In particular, the average correlations for the angular position and angular velocity are 0.9656 and 0.8375, respectively. In particular, the velocity states exhibit lower correlation coefficients than the position states concerning the actual experimental behavior, attributed to the noisy measurement in the velocity states. The noise in the identification tasks is a significant challenge, often necessitating well-tuned filtering techniques, advanced measurement instrumentation, or meticulous trajectory planning [77].

#### (ii) Model validation in closed-loop

In this step, two control strategies are also employed to test two different MiL simulation environments: 1) A PID control strategy for regulation at a single point in the joint space and 2) A PD+G control strategy for following a smooth trajectory in the joint space.

The comparisons between the model and the testbed platform, which include graphical representations and corresponding correlation coefficients, form a thorough validation process. This process instills confidence in the model’s accuracy by evaluating how well the model’s predicted states match the actual measured states. The comprehensive analysis aims to validate the model’s effectiveness in capturing the dynamics and behaviors of the testbed platform under different operational conditions.

This study’s primary validation measures are the comparisons between measured and estimated position and velocity states. However, additional validation measures, as suggested by [93], could include the prediction of the necessary torque in the actuators to achieve a specific task or how far the original model parameters (perhaps required from the manufacturer) lie against the predicted values (confidence interval). These validation measures typically require more complex and potentially expensive means of data collection and analysis. Therefore, while these measures are valuable for a comprehensive validation of the model’s predictive capability, they are not included in the scope of this particular study. The focus here remains on validating the positional and velocity states as a primary indicator of the model’s accuracy in representing the behavior of the testbed platform.

**Regulate a point in the joint space with the PID control strategy**

Table 15 presents the positions to be reached in the joint space for each degree of freedom and the PID controller parameters used for each joint, applicable to both the model and the laboratory testbed platform.

**Table 15:** PID controller parameters and the desired position in the joint space used in each d.o.f. for the validation of the obtained RRR robot model in the regulation task.

Link	Desired position	Kp	Kd	Ki
1	π2	200	20	0.8
2	$-\frac{\pi }{4}$	150	30	0.8
3	$-\frac{\pi }{4}$	110	0.3	0.6

**Fig 13:**
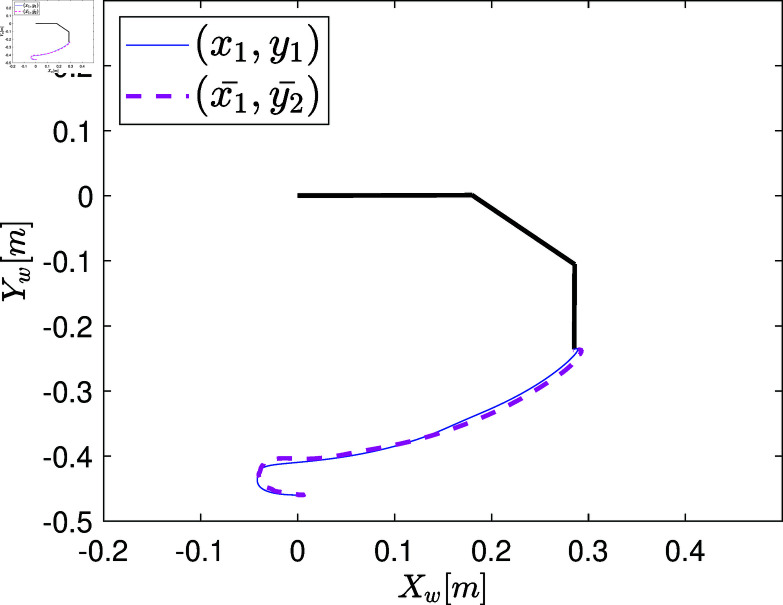
Robot’s end-effector behavior in the workspace $\left[X_{w},Y_{w}\right]$ of the obtained model against the real experiment for the regulation task using PID controller. Black continuous lines indicate the robot links.

Fig 13 illustrates the workspace of the manipulator $\left(X_{w},Y_{w}\right)$, where the thick black lines represent the robot’s links with the end effector at one end, the solid blue line shows the measured real values (x,y), and the thick magenta dotted line represents the predicted values $\left(\bar{x},\bar{y}\right)$.

**Fig 14:**
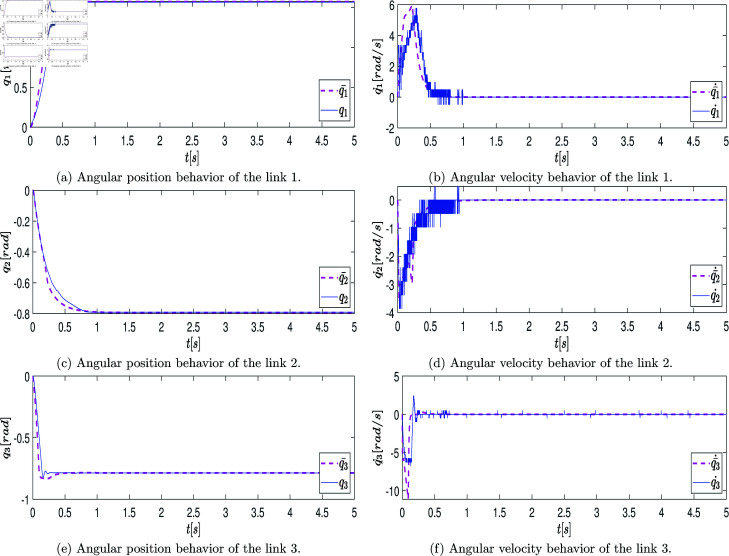
Angular position and velocity behaviors of the RRR robot in the regulation task using PID controller. Real experiment (*q*_1_, *q*_2_, *q*_3_, $\dot{q_{1}}$, $\dot{q_{2}}$, $\dot{q_{3}}$) vs. approximated model ($\bar{q_{1}}$, $\bar{q_{2}}$, $\bar{q_{3}}$, $\dot{\bar{q_{1}}}$, $\dot{\bar{q_{2}}}$, $\dot{\bar{q_{3}}}$) with the solution p^*^ obtained by PSO.

**Fig 15:**
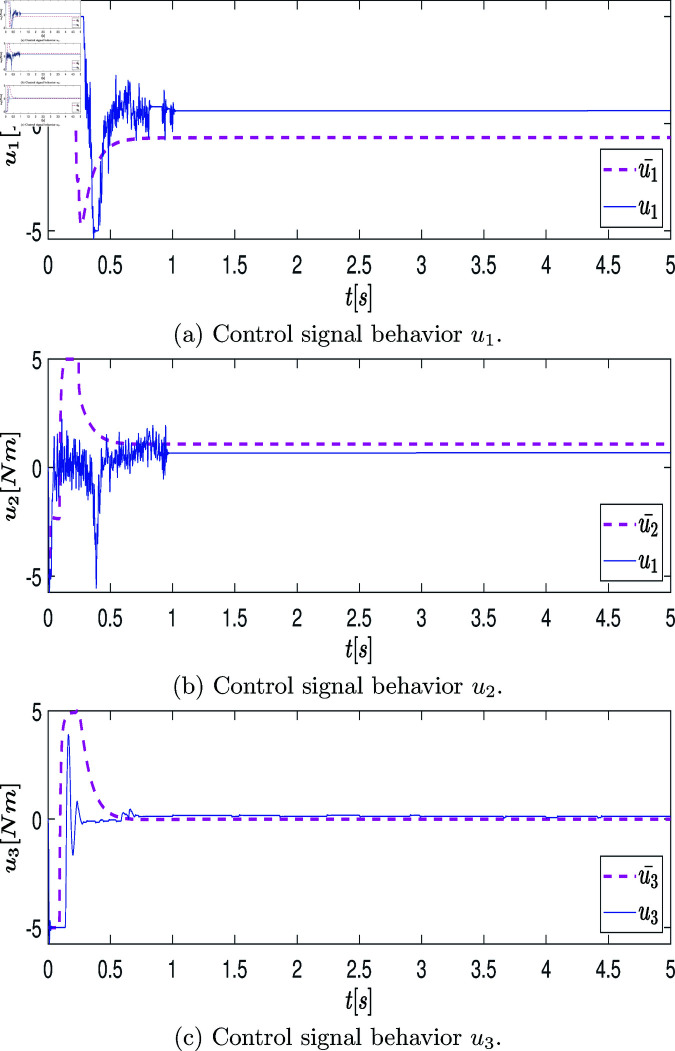
Control signals ($u_{1},u_{2},u_{3}$) for each d.o.f. obtained from the regulation PID control experiment. Real experiment (*u*_1_, *u*_2_, *u*_3_) vs. approximated model ($\bar{u_{1}}$, $\bar{u_{2}}$, $\bar{u_{3}}$) with the solution p^*^ obtained by PSO.

Concerning the controller performances, Fig 14a, 14c, 14e presents the actual and approximated position states $q_{1},q_{2},q_{3}$ for the regulation task. Likewise, Fig 14b, 14d, 14f includes the real and approximate velocity states $\dot{q_{1}},\dot{q_{2}},\dot{q_{3}}$ for the same task. The control signals applied to the model and testbed platform are presented in Fig 15.

Table 16 presents the respective correlation coefficients between the states of the testbed platform and those of the model for this experiment. Based on this table and the corresponding Fig 14 in the regulation task with the PID controller, it is observed that the overall correlations in the Cartesian space of the robot are very confident in the interval [0.9753,0.9871], meanwhile the angular position space presents a correlation in the interval [0.9480,0.9924] and the angular velocity space provides a correlation in the interval [0.7949,0.9467]. With this information, the approximated model successfully behaves as the robot’s end-effector behavior of the testbed platform.

**Table 16:** Correlation coefficient ρ between the obtained model signals and the robot signals in the regulation task with the PID controller (closed loop).

State	ρ
*q*_1_	0.986599969
*q*_2_	0.992442123
*q*_3_	0.948025947
$\dot{q_{1}}$	0.897757993
$\dot{q_{2}}$	0.946706468
$\dot{q_{3}}$	0.794907363
Cartesian workspace in *X*_*w*_	0.975326977
Cartesian workspace in *Y*_*w*_	0.987160562

**Smooth tracking of a path with the PD+G control strategy**

In this test, the trajectory consists of tracking three points in the joint space, executed in two different quadrants of the Cartesian space. Those points are joined by a third-order Bezier polynomial, resulting in a smooth trajectory. Table 17 displayed the points reached by this trajectory and the controller parameters used for the PD+G control in both the modeling and experimental setups.

**Fig 16:**
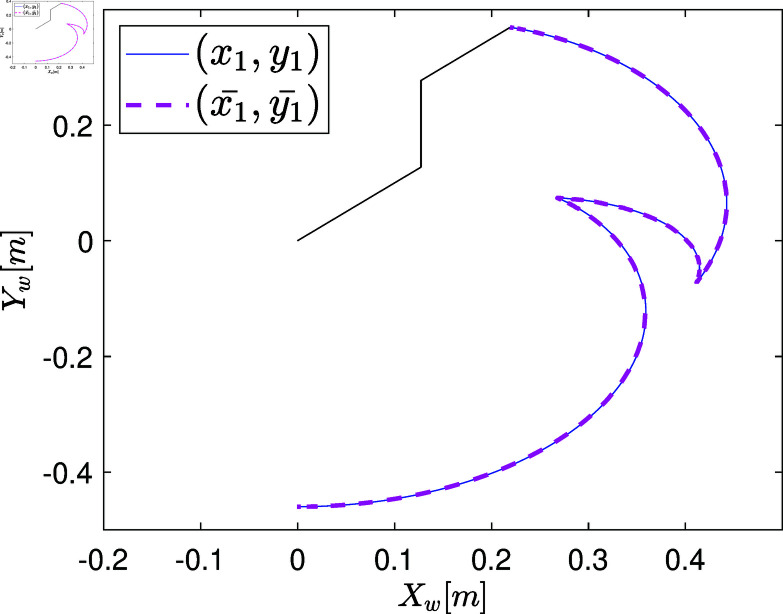
Robot’s behavior in the workspace $\left[X_{w},Y_{w}\right]$ of the obtained model against the real experiment for the tracking task using PD+G controller. Black continuous lines indicate the robot links.

Fig 16 presents a view of the manipulator’s workspace (X^w^,Y^W^), showing the comparison of both the real measured behavior in solid lines and the predicted one in dotted lines, following the convention of previous plots, but this time for the tracking task.

**Fig 17:**
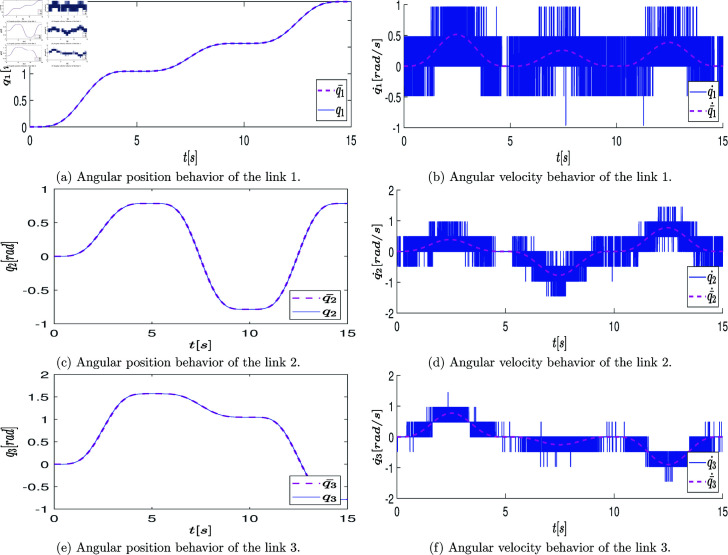
Angular position and velocity behaviors of the robot in the tracking task using PD+G controller. Real experiment ($q_{1}$, $q_{2}$, $q_{3}$, $\dot{q_{1}}$, $\dot{q_{2}}$, $\dot{q_{3}}$) vs. approximated model ($\bar{q_{1}}$, $\bar{q_{2}}$, $\bar{q_{3}}$, $\dot{\bar{q_{1}}}$, $\dot{\bar{q_{2}}}$, $\dot{\bar{q_{3}}}$) with the solution $p^{*}$ obtained by PSO.

**Fig 18:**
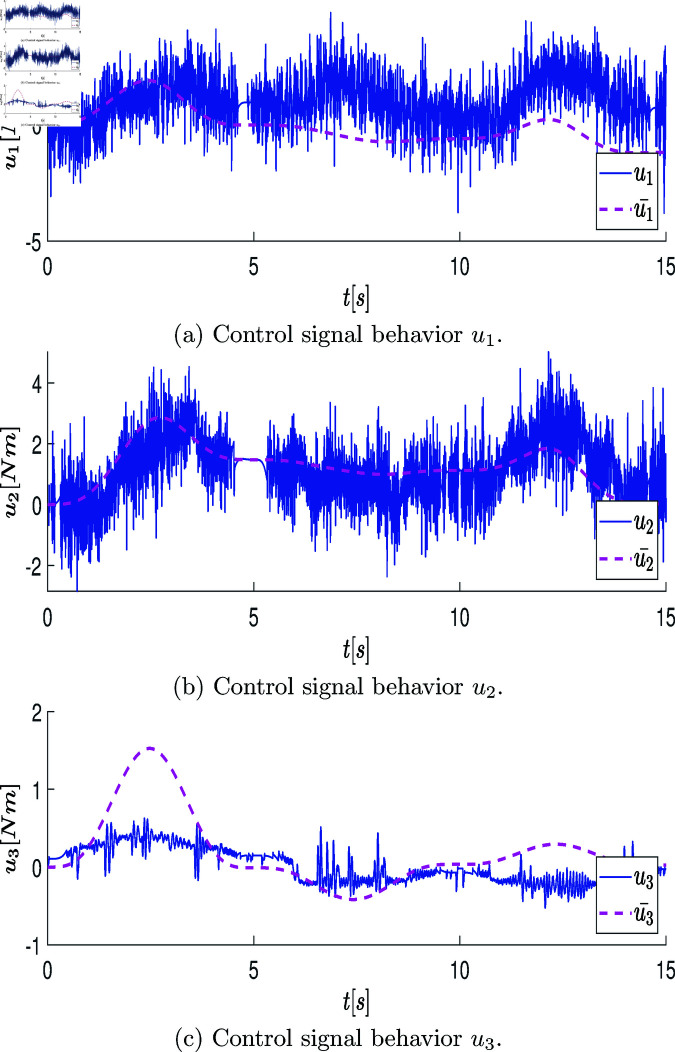
Control signals ($u_{1},u_{2},u_{3}$) for each d.o.f. obtained from the tracking PD+G control experiment. Real experiment (*u*_1_, *u*_2_, *u*_3_) vs. approximated model (${\bar{u}}_{1}$, ${\bar{u}}_{2}$, ${\bar{u}}_{3}$) with the solution p^*^ obtained by PSO.

The plots in Fig 17a, 17c, 17e present the real and approximate position states of $q_{1},q_{2},q_{3}$ for the tracking task. Similarly, the real and approximate velocity states $\dot{q_{1}},\dot{q_{2}},\dot{q_{3}}$ are depicted in Fig 17b, 17d, 17f for the same trajectory tracking task. The control signals of the model and the testbed platform can also be observed in Fig 18.

Table 18 presents the respective correlation coefficients between the states of the testbed platform and those of the model for this experiment. Based on this table and Fig 17 in the tracking task with the PD+G controller, it is observed that the overall correlations in the Cartesian space of the robot and the angular position space are very confident with a value around one. Meanwhile, the angular velocity space provides a correlation in the interval [0.5227,0.9009]. With this information, the approximated model successfully behaves as the robot’s end-effector behavior of the testbed platform.

#### Highlights of the validation results

The model validation in the open loop control indicates a high average correlation among all joint states of 0.9015, indicating a high representation of the real system with the obtained parameterized model. In particular, the average correlation in the angular position, angular velocity, and Cartesian position is 0.9656, 0.8375, and 0.8969, respectively.

On the other hand, the tests in the MiL simulation using the obtained parameterized model for the regulation and the tracking control problems (closed loop control) shows a high average correlation among all joint states in both control problems of 0.9022, revealing the model’s effectiveness for general-purpose use in MiL tests. In particular, the correlations given for the Cartesian space is in the interval [0.9753,1] and for the angular position space is in the interval [0.9480,1] providing an average correlation of 0.9906 and 0.9878, respectively. Meanwhile, the correlation in the angular velocity space is in the interval [0.5227,0.9467] with an average correlation of 0.8165. The slight decrement in the angular velocity correlation is attributed to the estimation of the angular velocity by using finite difference because it produces noise in the velocity measure and also in the trade-off selected in Stage IV.

### Application of the MbPBO methodology to the RFSEA

The main purpose of this section is to provide useful information about the applicability and the flexibility of the MbPBO methodology in different electro-mechanical systems. In this case, the proposed MbPBO methodology is applied to the model parameterization of the Reaction Force-sensing Series Elastic Actuator (RFSEA), confirming the similarities in the behavior of the obtained model with respect to the real system’s behavior. In what follows, some aspects of the methodology are highlighted.

The RFSEA is a series elastic linear actuator [105] where the tip of actuation (the ball screw) involves an active and passive movement through the displacement of the pulley-belt transmission and spring, respectively. The Series Elastic Actuator (SEA) has been widely used in robotic leg [106], biomechanical devices [107–109], mechatronic systems [110], lower and upper limb rehabilitation devices [111,112], among others. The most important aspects of the MbPBO methodology are highlighted next.

**Table 17:** PD+G controller parameters and the desired positions in the joint space joined by third-order Bezier polynomials for the validation of the obtained RRR robot model in the tracking task.

Link	Desired consecutive position	Kp	Kd
1	$\frac{\pi }{3},\frac{\pi }{2},\frac{3\pi }{4}$	2000	20
2	$\frac{\pi }{4},-\frac{\pi }{4},\frac{\pi }{4}$	2000	30
3	$\frac{\pi }{2},\frac{\pi }{3},-\frac{\pi }{4}$	110	0.3

**Fig 19:**
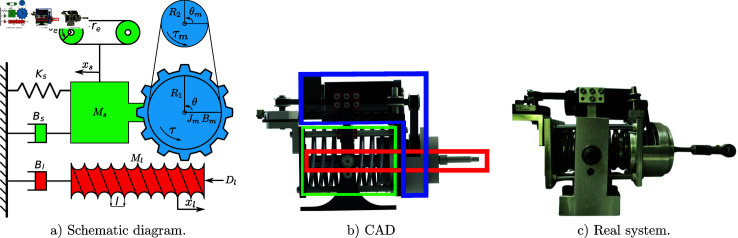
RFSEA system.

Fig 19 represents the RFSEA system divided into the three most important parts of movements in different colors: The motor’s belt-pulley transmission, the spring movement, and the linear motion of the ball screw. The variables associated in the schematic diagram of the RFSEA are: (A) For the belt-pulley transmission: the motor’s angular position $\theta_{m}$, the input torque $\tau_{m}$ (motor’s torque), the output torque τ, the motor’s current *i*_*a*_, the motor’s inertia *J*_*m*_, the motor’s damping coefficient *B*_*m*_ and the radii of the drive pulleys *R*_1_ and *R*2. (B) For the spring movement, the spring’s linear displacement *x*_*s*_, the spring’s stiffness coefficient *K*_*s*_, the spring’s mass *M*_*s*_, the spring’s damping coefficient *B*_*s*_, the pulley radius *r*_*e*_ for the linear spring’s displacement and its angular displacement θ_e_. (C) For the ball screw’s linear motion, the ball screw displacement *x*_*l*_, the ball screw’s damping coefficient *B*_*l*_, the ball screw’s mass *M*_*l*_, the screw pitch *l* and the load’s reaction force *D*_*l*_.

The dynamic equations of the RFSEA consist of the mechanical (37)-(39) and electrical (40) dynamics [113–115], where $N_{m}=\frac{2\pi N_{p}}{l}$ is the transmission ratio of the ball screw with $N_{p}=\frac{R_{1}}{R_{2}}$ as the belt-pulley transmission ratio obtained when the larger pulley with radius *R*_1_ is the output and the smaller pulley with radius *R*_2_ is the input.

$$M_{s}{\ddot{x}}_{s}+K_{s}x_{s}-\lambda +B_{s}{\dot{x}}_{s}=0$$ (37)

$$M_{l}\ddot{x_{l}}+\lambda +B_{l}\dot{x_{l}}=F_{l}$$ (38)

$$J_{m}\ddot{\theta_{m}}-\lambda N_{m}^{-1}+B_{m}\dot{\theta_{m}}=\tau_{m}$$ (39)

$$L_{a}\frac{di_{a}}{dt}+R_{a}i_{a}+K_{e}{\dot{\theta }}_{m}=V_{in}$$ (40)

The dynamic coupling of the mechanical and electrical parts of the RFSEA assuming $\tau_{m}=K_{m}i_{a}$ can be expressed in state space $x=[x_{1},x_{2},x_{3},x_{4},x_{5}]=[x_{l},x_{s},{\dot{x}}_{l},{\dot{x}}_{s},i_{a}{]}^{T}\in {\mathbb{R}}^{5}$ with the input signal $u=V_{in}\in \mathbb{R}$ and the reaction force *D*_*l*_. The state-space representation of the RFSEA dynamic model results in (41), where $\beta_{0}=-(M_{l}M_{s}+J_{m}M_{l}N_{m}^{2}+J_{m}N_{m}^{2}M_{s})/(J_{m}^{2}N_{m}^{4})$, $\beta_{1}=K_{s}/(J_{m}N_{m}^{2})$, $\beta_{5}=-(M_{s}$  +  $J_{m}N_{m}^{2})/(J_{m}N_{m}^{2}{)}^{2}$, $\beta_{2}=(B_{l}M_{s}$  +  $B_{l}J_{m}N_{m}^{2}$  +  $B_{m}N_{m}^{2}M_{s})/(J_{m}^{2}N_{m}^{4})$, $\beta_{3}=(B_{s}J_{m}$ − $B_{m}M_{s})/(J_{m}^{2}N_{m}^{2})$, $\beta_{4}=-K_{m}M_{s}/(J_{m}^{2}N_{m}^{3})$, $\beta_{6}=K_{s}(J_{m}N_{m}^{2}$  +  $M_{l})/(J_{m}^{2}N_{m}^{4})$, $\beta_{7}=(B_{l}J_{m}$ − $B_{m}M_{l})/(J_{m}^{2}N_{m}^{2})$, $\beta_{8}=(B_{s}M_{l}$  +  $B_{m}M_{l}N_{m}^{2}$  +  $B_{s}J_{m}N_{m}^{2})/(J_{m}^{2}N_{m}^{4})$, $\beta_{9}=K_{m}M_{l}/(J_{m}^{2}N_{m}^{3})$, $\beta_{10}=-1/(J_{m}N_{m}^{2})$, $\beta_{11}=-K_{e}N_{m}/L_{a}$, $\beta_{12}=K_{e}N_{m}/L_{a}$, $\beta_{13}=-R_{a}/L_{a}$ and $\beta_{14}=1/L_{a}$.

$$\dot{x}(t)=Ax(t)+Bu(t)+ED_{l}$$ (41)

where:


$$A=[\begin{array}{ccccc} 0 & 0 & 1 & 0 & 0\\ 0 & 0 & 0 & 1 & 0\\ 0 & \beta_{1}/\beta_{0} & \beta_{2}/\beta_{0} & \beta_{3}/\beta_{0} & \beta_{4}/\beta_{0}\\ 0 & \beta_{6}/\beta_{0} & \beta_{7}/\beta_{0} & \beta_{8}/\beta_{0} & \beta_{9}/\beta_{0}\\ 0 & 0 & \beta_{11} & \beta_{12} & \beta_{13} \end{array}],\;B=[\begin{array}{c} 0\\ 0\\ 0\\ 0\\ \beta_{14} \end{array}],$$

$$E=[\begin{array}{c} 0\\ 0\\ \beta_{5}/\beta_{0}\\ \beta_{10}/\beta_{0}\\ 0 \end{array}],\;C=[\begin{array}{ccccc} 1 & 0 & 0 & 0 & 0\\ 0 & 1 & 0 & 0 & 0\\ 0 & 0 & 0 & 0 & 1 \end{array}]$$

**Table 18:** Correlation coefficient ρ between the obtained model signals and the robot signals in the tracking task with the PD+G controller (closed loop).

State	ρ
*q*_1_	0.999999475
*q*_2_	0.999997738
*q*_3_	0.999990784
q˙^1^	0.522790413
$\dot{q_{2}}$	0.836160822
$\dot{q_{3}}$	0.900984464
Cartesian workspace in *X*_*w*_	0.999995562
Cartesian workspace in *Y*_*w*_	0.999997765

The Stage I is finished with the obtained model of the RFSEA. The characterization of the system’s behavior in Stage II is carried out in the same manner as described in the MbPBO methodology. The specific set of parameters to be found and the objective function are selected in the optimization process for Stage III. In this case, the selected design parameters vector is related to the damping coefficients $𝐩=[B_{m},B_{s},B_{l}]\in {\mathbb{R}}^{3}$ with the terms of the objective functions as $J_{i}=\int_{0}^{t_{f}}(x_{i}-{\bar{x}}_{i}{)}^{T}(x_{i}-{\bar{x}}_{i})\;dt$, where ${\bar{x}}_{i}$ is the estimated state. In addition, the normalization is done with the corresponding maximum values of the objective function terms obtained in the procedure (J1MAX=1.54×10−6, J2MAX=2.25×10−8, J3MAX=19.39×10−5, J4MAX=5.73 × 10^−5^, J5MAX=252.85). The values of the rest model parameters associated to the RFSEA are described through the datasheet of components, or by directly using measuring instruments. Following Stage IV of the proposed model parameterization approach, the selected weights are $\mu_{1}=\mu_{2}=\mu_{3}=\mu_{4}=\mu_{5}=0.2$, giving the same importance to all terms in the objective function. In Table 19, the parameters obtained using the MbPBO optimization process are indicated with an asterisk, along with the remaining parameters employed.

**Table 19:** Parameters of the RFSEA.

Parameter	Value	Parameter	Value
*K*_*s*_	33861 *N*/*m*	*L*_*a*_	0.0820 *mH*
*M*_*l*_	0.079 *kg*	*K*_*m*_	0.0302 *Nm*/*A*
*M*_*s*_	0.0797 *kg*	*K*_*e*_	0.0301 V/rad/s
*N*_*m*_	9817.47	$B_{m}^{*}$	$1.06\times 10^{-4}$ *Nms*/*rad*
*J*_*m*_	$1.42\times 10^{-5}$ *kgm*^2^	$B_{s}^{*}$	218.52421 *Ns*/*m*
*R*_*e*_	0.2990 Ω	$B_{l}^{*}$	16.50665 *Ns*/*m*

**Fig 20:**
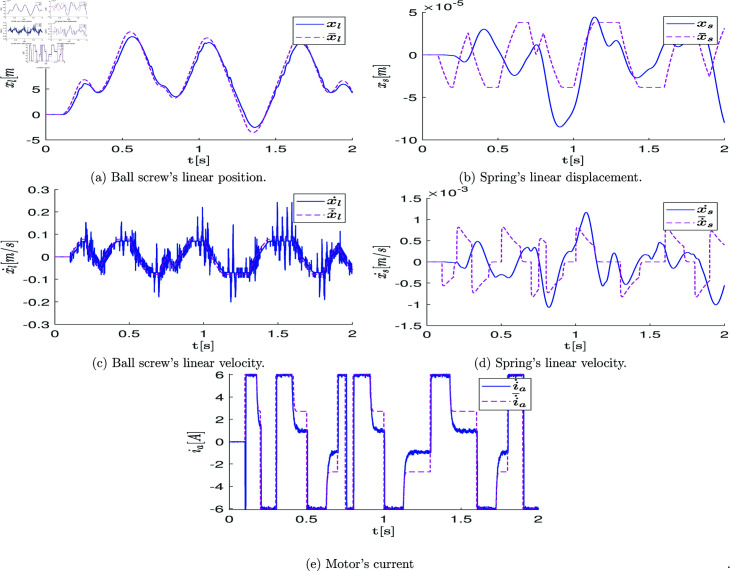
State behaviors of the obtained RFSEA model against the real experiment signals when the system states are excited by the open-loop control signals. Real experiment (*x*_*l*_, *x*_*s*_, ${\dot{x}}_{l}$, ${\dot{x}}_{s}$, *i*_*a*_) vs. approximated model (${\bar{x}}_{l}$, ${\bar{x}}_{s}$, ${\dot{\bar{x}}}_{l}$, ${\dot{\bar{x}}}_{s}$, ${\bar{i}}_{a}$).

With the obtained parameters shown in Table 19, the parameterized model is completed, and the validation of the obtained parameterized model using an open-loop controller is carried out as described in Stage V. In this case, the drivers to control the RFSEA motor are configured in voltage mode, so the reference signals in the open-loop controller are related to the voltage $V_{in}$ and as a consequence the open-loop control signal is *u* = *ref* (a cascade controller is not applied in the RFSEA case). The behaviors of the state vectors for the real experiment and the approximated model with the same persistent excitation control signal are given in Fig 20. The bar above the variable indicates the behavior of the obtained model. The Pearson correlation coefficients of the signals related to the screw displacement, the screw velocity, and the current present a suitable correlation of 0.9919,0.8771, and 0.9226, respectively. Those correlations indicate that the obtained model represents a similar behavior to the real system. The worst correlation coefficients are given by the spring position and spring velocity (ρ=0.3399 and ρ=0.0765, respectively). This is attributed because those measures present small magnitudes (see Fig 20b, 20d) due to spring states are not excited in the experiments by applying the external force *D*_*l*_ and are mainly affected by the vibrations of the testbed platform. So, those spring measures are not representative in the study.

**Fig 21:**
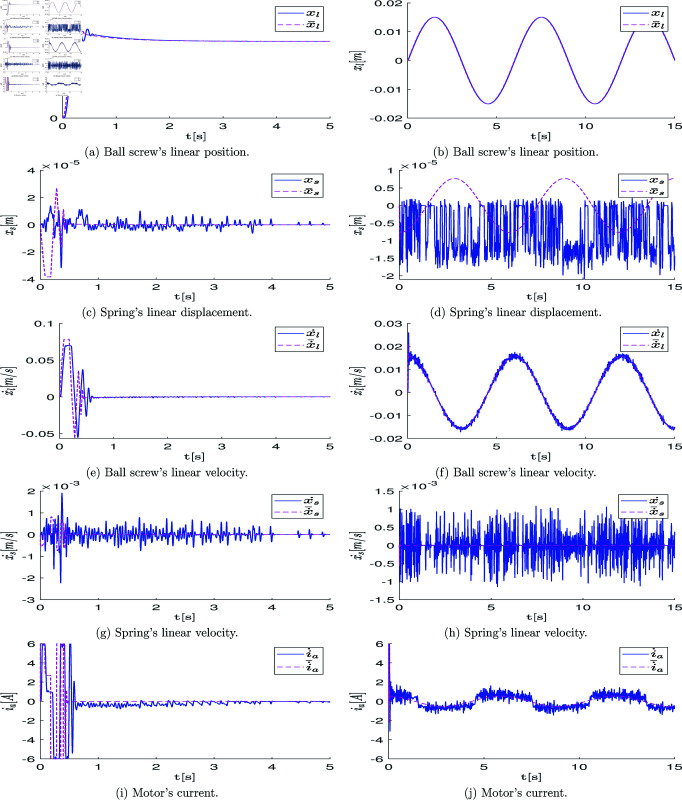
Behavior of the RFSEA’s states in the regulation (left) and tracking (right) tasks. Real experiment (*x*_*l*_, *x*_*s*_, ${\dot{x}}_{l}$, ${\dot{x}}_{s}$, *i*_*a*_) vs. approximated model (${\bar{x}}_{l}$, ${\bar{x}}_{s}$, ${\dot{\bar{x}}}_{l}$, ${\dot{\bar{x}}}_{s}$, ${\bar{i}}_{a}$).

On the other hand, the validation of the obtained parameterized model using a closed-loop controller for testing two different MiL simulation environments is carried out as described in Stage V. Table 20 provides the respective correlation coefficients between the states of the RFSEA testbed platform and those of the model for the regulation and tracking tasks. The representative figures for those signals are presented in Fig 21. Based on this table, it is observed that the average correlation in the regulation and tracking tasks in the ball screw’s linear displacement is 0.9743 and in the ball screw’s linear velocity is 0.8356, indicating high correlation values, i.e., the model data are highly related to the experimental data using the same input data, meaning that the model is useful for describing the real behavior of those signals. In the motor’s current, the average correlation is 0.4263, indicating a moderate correlation value and that the obtained model behavior could present deviations from the real behavior, which is mainly attributed to a low current transducer sensitivity. In the case of the spring position and its velocity, these signals present an average correlation of −0.1749 and 0.0446, respectively. Those correlation values indicate that there is no relationship between the model data and the real behavior. Nevertheless, those weak correlations are attributed to the spring signals not being excited in either the model or the experimentation. So, their movements in the experimental data have small magnitudes (see Fig 21c, 21d, 21g, 21h) mainly given by the vibrations of the testbed platform which are not considered in the model. For instance, the movement of the spring is not perturbed by an external load in both experiments. Then, the spring position and velocity correlations are not representative in the study. So, the results obtained in the MiL simulation environments indicate that the obtained RFSEA model can describe, to a large extent, the real behavior.

**Table 20:** Correlation coefficient ρ between the obtained model signals and the system signals in the regulation and tracking tasks with the PID controller in the RFSEA.

State	Regulation task	Tracking task
	ρ	ρ
*x*_*l*_	0.9488	0.9998
*x*_*s*_	−0.1514	−0.1985
${\dot{x}}_{l}$	0.6751	0.9960
${\dot{x}}_{s}$	0.1097	−0.0205
*i*_*a*_	0.1499	0.7027

### General discussion

Based on the outcomes achieved at various stages of applying the MbPBO methodology, the following is highlighted:

- Bio-inspired techniques are widely regarded as effective alternatives for modeling and identification tasks. Based on a comparative analysis involving PSO, GA, and DE algorithms in solving optimization problems, the PSO algorithm consistently converges toward the best solution across multiple executions.
- A persistent pattern is observed in the percentage variation between the nominal values of the experiment and the solutions obtained using the three algorithms. These findings underscore the importance of the MbPBO methodology in accurately parametrizing models to achieve behavior closer to real systems. Despite unmodeled uncertainties in the investigation, such as noise signal, backlash between gears, and hysteresis, among others, the simulated states exhibit a high average general correlation of 0.9019 considering open-loop control and various tasks and control strategies, including regulation and tracking tasks (MiL tests) in the joint space. Particularly, this model achieves an average correlation of 0.9015 and 0.9022 in open and closed-loop control, respectively. This high positive correlation indicates that the behavior of the obtained model is strongly related to the behavior of the real robotic system. This means that the obtained model can be used to predict the system’s behavior.
- The MbPBO methodology applied to a different electro-mechanical system (RFSEA) reveals the flexibility of the proposal. In this case, considering the states that were excited in the RFSEA, the correlations in open and closed loop control are 0.9305 and 0.7454, providing a high average general correlation of 0.8379. This correlation indicates a suitable approximated model.
- Obtaining a representative system model that closely approximates reality offers several advantages. Firstly, it eliminates the need for repetitive tests on the actual system when maximizing task performance, such as in controller tuning. Secondly, the methodology includes model validation within the MiL approach, accelerating the implementation of model-based design (MiL-SiL-HiL).

## Conclusions

This paper proposes the Model-Based Parametric Bioinspired Optimization (MbPBO) methodology. The proposal utilizes a nominal model based on mathematical relationships describing the system and identifies specific parameters through a bio-inspired optimization process framed as an identification experiment. The MbPBO methodology aims to develop an effective model for predicting the behavior of robotic systems integrated within the MiL simulation framework. The MbPBO methodology can also be applied to different systems, revealing a suitable obtained model when applied to the RFSEA electro-mechanical system.

The application of MbPBO methodology to a three-degree-of-freedom manipulator robot case study demonstrates that the methodology produces a parametrized model with a high average general correlation of 0.9019 considering open-loop control and closed-loop control strategies (MiL tests). When the MbPBO methodology is applied to the RFSEA, a high average general correlation of 0.8379 is obtained. Those results validate the parametrized model’s effectiveness and methodology, showing the model’s capacity to accurately forecast the behavior of the system and its strong correlation with the real system.

The parametrized model obtained through MbPBO methodology enhances the efficiency of the model-based design cycle (MiL-SiL-HiL). Additionally, it facilitates iterative adjustments or learning processes to optimize the performance of robotic systems without requiring direct interaction with the physical system.

Future research consists of developing virtual laboratories for robotic systems, where the MiL test will be applied to validate the controller design and the optimal tuning of its parameters. Virtual laboratories provide a controlled, cost-effective environment for testing and optimizing robotic control strategies through high-fidelity simulations. They reduce risks and expenses by allowing researchers to refine controller parameters and detect failures before real-world deployment. Additionally, these labs enable remote collaboration, scalability, and experimentation across different scenarios, fostering advancements in adaptive control, human-robot interaction, and reinforcement learning-based optimizations to enhance robotic system efficiency and reliability.

## References

[1] Nekoo SR, Acosta JÁ, Heredia G, Ollero A. A benchmark mechatronics platform to assess the inspection around pipes with variable pitch quadrotor for industrial sites. Mechatronics. 2021;79:102641. doi: 10.1016/j.mechatronics.2021.102641

[2] Malkowsky S, Vieira J, Liu L, Harris P, Nieman K, Kundargi N, et al. The world’s first real-time testbed for massive MIMO: design, implementation, and validation. IEEE Access. 2017;5:9073–88. doi: 10.1109/access.2017.2705561

[3] Tarn TJ, Shoults GA, Yang SP. A dynamic model of an underwater vehicle with a robotic manipulator using Kane’s method. Auton Robot. 1996;3(2–3):269–83. doi: 10.1007/bf00141159

[4] Parsi SS, Sivaselvan MV, Whittaker AS. Impedance-matching model-in-the-loop simulation. Earthq Eng Struct Dyn. 2023;52(12):3600–21. doi: 10.1002/eqe.3922

[5] Jingjing L, Shuai X, Jiawei L, Shijun Z, Chi Z. Design and in-orbit application of a new generalized fully-configured digital satellite based on software-in-the-loop. J Phys Conf Ser. 2022;2352(1):012009. doi: 10.1088/1742-6596/2352/1/012009

[6] Ayachi Amor Y, Hamoudi F, Kheldoun A, Didier G, Rabiai Z. Fuzzy logic enhanced control for a single-stage grid-tied photovoltaic system with shunt active filtering capability. Int Trans Electr Energy Syst. 2021;31(10). doi: 10.1002/2050-7038.13008

[7] Gambier A. Real-time control and hardware-in-the-loop simulation for educational purposes of wind energy systems. IFAC-PapersOnLine. 2020;53(2):17344–9. doi: 10.1016/j.ifacol.2020.12.2084

[8] Liu J, Gao F, Liu H, Li P. Multi-metrics restricts on MPRs over standard OLSR. In: 2015 Fifth International Conference on Instrumentation and Measurement, Computer, Communication and Control (IMCCC). IEEE; 2015, pp. 364–8.

[9] Ljung L. System identification: theory for the user. Prentice Hall PTR; 1999.

[10] Sutulo S, Guedes Soares C. An algorithm for offline identification of ship manoeuvring mathematical models from free-running tests. Oce Eng. 2014;79:10–25. doi: 10.1016/j.oceaneng.2014.01.007

[11] Villarreal-Cervantes MG, Alvarez-Gallegos J. Off-line PID control tuning for a planar parallel robot using DE variants. Expert Syst Appl. 2016;64:444–54. doi: 10.1016/j.eswa.2016.08.013

[12] Rodríguez-Molina A, Villarreal-Cervantes MG, Pantoja-García JS, Rojas-López AG, Hernández-Castillo E, Mejía-Rodríguez R. Metaheuristic adaptive control based on polynomial regression and differential evolution for robotic manipulators. Appl Soft Comput. 2024;151:111116. doi: 10.1016/j.asoc.2023.111116

[13] Dolatabadi PD, Khanlari K, Ghafory Ashtiany M, Hosseini M. System identification method by using inverse solution of equations of motion in time domain and noisy condition. Phys A Stat Mech Appl. 2020;538:122680. doi: 10.1016/j.physa.2019.122680

[14] Xu K-F, Zhang Y-W, Niu M-Q, Chen L-Q. Dynamics analysis of a variable stiffness tuned mass damper enhanced by an inerter. Appl Sci. 2023;13(3):1404. doi: 10.3390/app13031404

[15] McDonald DQ, Vallett R, Solovey E, Dion G, Shokoufandeh A. Knitted sensors. Proc ACM Interact Mob Wearable Ubiquitous Technol. 2020;4(4):1–25. doi: 10.1145/343220135846237

[16] Chen D, Seborg DE. Robust Nyquist array analysis based on uncertainty descriptions from system identification. Automatica. 2002;38(3):467–75. doi: 10.1016/s0005-1098(01)00207-2

[17] Leboutet Q, Roux J, Janot A, Guadarrama-Olvera JR, Cheng G. Inertial parameter identification in robotics: a survey. Appl Sci. 2021;11(9):4303. doi: 10.3390/app11094303

[18] Kommenda M, Burlacu B, Kronberger G, Affenzeller M. Parameter identification for symbolic regression using nonlinear least squares. Genet Program Evolvable Mach. 2019;21(3):471–501. doi: 10.1007/s10710-019-09371-3

[19] Ballesteros M, Polyakov A, Efimov D, Chairez I, Poznyak AS. Non-parametric identification of homogeneous dynamical systems. Automatica. 2021;129:109600. doi: 10.1016/j.automatica.2021.109600

[20] Bhowmik B, Tripura T, Hazra B, Pakrashi V. Real time structural modal identification using recursive canonical correlation analysis and application towards online structural damage detection. J Sound Vib. 2020;468:115101. doi: 10.1016/j.jsv.2019.115101

[21] Gedon D, Wahlström N, Schön TB, Ljung L. Deep state space models for nonlinear system identification. IFAC-PapersOnLine. 2021;54(7):481–6. doi: 10.1016/j.ifacol.2021.08.406

[22] Jansson M. Subspace identification and ARX modeling. IFAC Proc Vol. 2003;36(16):1585–90. doi: 10.1016/s1474-6670(17)34986-8

[23] Vidal R. Recursive identification of switched ARX systems. Automatica. 2008;44(9):2274–87. doi: 10.1016/j.automatica.2008.01.025

[24] Piroddi L, Spinelli W. An identification algorithm for polynomial NARX models based on simulation error minimization. Int J Control. 2003;76(17):1767–81. doi: 10.1080/00207170310001635419

[25] Moonen M, De Moor B, Vandenberghe L, Vandewalle J. On- and off-line identification of linear state-space models. Int J Control. 1989;49(1):219–32. doi: 10.1080/00207178908559631

[26] Schön TB, Wills A, Ninness B. System identification of nonlinear state-space models. Automatica. 2011;47(1):39–49. doi: 10.1016/j.automatica.2010.10.013

[27] Adigintla S, Aware MV, Arun N. Fractional order transfer function identification of six-phase induction motor using dual-chirp signal. IEEE J Emerg Sel Topics Power Electron. 2023;11(5):5183–94. doi: 10.1109/jestpe.2023.3296904

[28] Hachino T, Takata H. Identification of continuous-time nonlinear systems by using a Gaussian process model. IEEJ Trans Electr Electron Eng. 2008;3(6):620–8. doi: 10.1002/tee.20323

[29] Liu GP. Nonlinear identification and control: a neural network approach. Springer Science & Business Media; 2012.

[30] Elisei-Iliescu C, Dogariu L-M, Paleologu C, Benesty J, Enescu A-A, Ciochină S. A Recursive least-squares algorithm for the identification of trilinear forms. Algorithms. 2020;13(6):135. doi: 10.3390/a13060135

[31] Long B, Zhu Z, Yang W, Chong KT, Rodriguez J, Guerrero JM. Gradient descent optimization based parameter identification for FCS-MPC control of LCL-type grid connected converter. IEEE Trans Ind Electron. 2022;69(3):2631–43. doi: 10.1109/tie.2021.3063867

[32] Yang B, Zeng C, Wang L, Guo Y, Chen G, Guo Z, et al. Parameter identification of proton exchange membrane fuel cell via Levenberg-Marquardt backpropagation algorithm. Int J Hydrogen Energy. 2021;46(44):22998–3012. doi: 10.1016/j.ijhydene.2021.04.130

[33] Rezk H, Olabi AG, Wilberforce T, Sayed ET. A comprehensive review and application of metaheuristics in solving the optimal parameter identification problems. Sustainability. 2023;15(7):5732. doi: 10.3390/su15075732

[34] Sachan S, Swarnkar P. Intelligent fractional-order sliding mode optimised control of surgical manipulator for healthcare system. Electr Eng. 2023;106(2):2131–42. doi: 10.1007/s00202-023-02052-6

[35] Sreekanth N, Dinesan A, Nair AR, Udupa G, Tirumaladass V. Design of robotic manipulator for space applications. Mater Today Proc. 2021;46:4962–70. doi: 10.1016/j.matpr.2020.10.382

[36] Yu D. Kinematic parameter identification for a parallel robot with an improved particle swarm optimization algorithm. Appl Sci. 2024;14(15):6557. doi: 10.3390/app14156557

[37] Lo W-L, Chung HS-H, Hsung RT-C, Fu H, Shen T-W. PV panel model parameter estimation by using particle swarm optimization and artificial neural network. Sensors (Basel). 2024;24(10):3006. doi: 10.3390/s24103006 38793862 PMC11125583

[38] Mellit A, Benghanem M, Kalogirou SA. Modeling and simulation of a stand-alone photovoltaic system using an adaptive artificial neural network: proposition for a new sizing procedure. Renew Energy. 2007;32(2):285–313. doi: 10.1016/j.renene.2006.01.002

[39] Kennedy J, Eberhart R. Particle swarm optimization. In: Proceedings of ICNN’95 - International Conference on Neural Networks, vol. 4. IEEE; 1995, pp. 1942–8. doi: 10.1109/icnn.1995.488968

[40] Martinelli C, Coraddu A, Cammarano A. Experimental parameter identification of nonlinear mechanical systems via meta-heuristic optimisation methods. In: Society for Experimental Mechanics Annual Conference and Exposition. Springer; 2023, pp. 215–23.

[41] Holland JH. Genetic algorithms. Sci Am. 1992;267(1):66–72. doi: 10.1038/scientificamerican0792-661411454

[42] Karahan O, Karci H. Swarm intelligence based nonlinear friction and dynamic parameters identification for a 6-DOF robotic manipulator. J Intell Robot Syst. 2023;108(2). doi: 10.1007/s10846-023-01868-5

[43] Yang XS, Deb S. Cuckoo search via Lévy flights. In: 2009 World Congress on Nature & Biologically Inspired Computing (NaBIC). IEEE; 2009, pp. 210–4.

[44] Mirjalili S, Mirjalili SM, Lewis A. Grey wolf optimizer. Adv Eng Softw. 2014;69:46–61. doi: 10.1016/j.advengsoft.2013.12.007

[45] Şenel FA, Gökçe F, Yüksel AS, Yiğit T. A novel hybrid PSO–GWO algorithm for optimization problems. Eng Comput. 2018;35(4):1359–73. doi: 10.1007/s00366-018-0668-5

[46] Rodríguez-Abreo O, Rodríguez-Reséndiz J, Álvarez-Alvarado JM, García-Cerezo A. Metaheuristic parameter identification of motors using dynamic response relations. Sensors (Basel). 2022;22(11):4050. doi: 10.3390/s22114050 35684670 PMC9185292

[47] Pandey HM. Jaya a novel optimization algorithm: What, how and why? In: 2016 6th International Conference - Cloud System and Big Data Engineering (Confluence). IEEE; 2016, pp. 728–30. doi: 10.1109/confluence.2016.7508215

[48] Yu X, Wu X, Luo W. Parameter identification of photovoltaic models by hybrid adaptive JAYA algorithm. Mathematics. 2022;10(2):183. doi: 10.3390/math10020183

[49] Danaci M, Koylu F, Al-Sumaidaee ZA. Identification of dynamic models by using metaheuristic algorithms. ADI J Recent Innovation (AJRI). 2021;3(1):36–58. doi: 10.34306/ajri.v3i1.492

[50] Simon D. Biogeography-based optimization. IEEE Trans Evol Comput. 2008;12(6):702–13. doi: 10.1109/tevc.2008.919004

[51] Dorigo M, Maniezzo V, Colorni A. Ant system: optimization by a colony of cooperating agents. IEEE Trans Syst Man Cybern B Cybern. 1996;26(1):29–41. doi: 10.1109/3477.484436 18263004

[52] Karaboga D. An idea based on honey bee swarm for numerical optimization. Erciyes University, Engineering Faculty, Computer; 2005.

[53] Rodriguez-Abreo O, Hernandez-Paredes JM, Rangel AF, Fuentes-Silva C, Velasquez FAC. Parameter identification of motors by cuckoo search using steady-state relations. IEEE Access. 2021;9:72017–24. doi: 10.1109/access.2021.3078578

[54] Ghafil HN, Jármai K. Dynamic differential annealed optimization: new metaheuristic optimization algorithm for engineering applications. Appl Soft Comput. 2020;93:106392. doi: 10.1016/j.asoc.2020.106392

[55] Aydilek İB. A hybrid firefly and particle swarm optimization algorithm for computationally expensive numerical problems. Appl Soft Comput. 2018;66:232–49. doi: 10.1016/j.asoc.2018.02.025

[56] Yang XS. Nature-inspired metaheuristic algorithms. Luniver Press; 2010.

[57] Mirjalili S. The ant lion optimizer. Adv Eng Softw. 2015;83:80–98. doi: 10.1016/j.advengsoft.2015.01.010

[58] Heidari AA, Mirjalili S, Faris H, Aljarah I, Mafarja M, Chen H. Harris hawks optimization: algorithm and applications. Future Gener Comput Syst. 2019;97:849–72. doi: 10.1016/j.future.2019.02.028

[59] Arora S, Singh S. Butterfly optimization algorithm: a novel approach for global optimization. Soft Comput. 2018;23(3):715–34. doi: 10.1007/s00500-018-3102-4

[60] Mirjalili S. SCA: a sine cosine algorithm for solving optimization problems. Knowl-Based Syst. 2016;96:120–33. doi: 10.1016/j.knosys.2015.12.022

[61] Mirjalili S, Lewis A. The whale optimization algorithm. Adv Eng Softw. 2016;95:51–67. doi: 10.1016/j.advengsoft.2016.01.008

[62] Alipour H, Abbaspour M, Esmaeili M, Mousavi H, Shahhoseini H. DACA: Dynamic Advanced Clustering Algorithm for Sensor Networks. In: 2007 14th IEEE International Conference on Electronics, Circuits and Systems. IEEE; 2007, pp. 518–25. doi: 10.1109/icecs.2007.4511043

[63] Cuevas E, Gálvez J, Avalos O. Recent metaheuristics algorithms for parameter identification. Springer; 2020.

[64] Rashedi E, Nezamabadi-pour H, Saryazdi S. GSA: a gravitational search algorithm. Inf Sci. 2009;179(13):2232–48. doi: 10.1016/j.ins.2009.03.004

[65] Storn R, Price K. Differential evolution–a simple and efficient heuristic for global optimization over continuous spaces. J Glob Optim. 1997;11(4):341–59. doi: 10.1023/a:1008202821328

[66] Askarzadeh A. A novel metaheuristic method for solving constrained engineering optimization problems: crow search algorithm. Comput Struct. 2016;169:1–12. doi: 10.1016/j.compstruc.2016.03.001

[67] Liu Y, Hou Z, Wang H, Liang J, Yang G, Zhang Y, et al. Parameter identification of collaborative robot based on improved artificial fish swarm algorithm. In: 2020 International Conference on High Performance Big Data and Intelligent Systems (HPBD&IS). IEEE; 2020, pp. 1–7. doi: 10.1109/hpbdis49115.2020.9130566

[68] Wang C, Zhou C, Ma J. An improved artificial fish-swarm algorithm and its application in feed-forward neural networks. In: 2005 International Conference on Machine Learning and Cybernetics. IEEE; 2005, pp. 2890–4.

[69] Wu Z, Shen D, Shang M, Qi S. Parameter identification of single-phase inverter based on improved moth flame optimization algorithm. Electr Power Compon Syst. 2019;47(4–5):456–69. doi: 10.1080/15325008.2019.1607922

[70] Mirjalili S. Moth-flame optimization algorithm: a novel nature-inspired heuristic paradigm. Knowl-Based Syst. 2015;89:228–49. doi: 10.1016/j.knosys.2015.07.006

[71] Urrea C, Pascal J. Design, simulation, comparison and evaluation of parameter identification methods for an industrial robot. Comput Electr Eng. 2018;67:791–806. doi: 10.1016/j.compeleceng.2016.09.004

[72] Zheng Y, Liao Y. Parameter identification of nonlinear dynamic systems using an improved particle swarm optimization. Optik. 2016;127(19):7865–74. doi: 10.1016/j.ijleo.2016.05.145

[73] Al-Dabbagh RD, Kinsheel A, Mekhilef S, Baba MS, Shamshirband S. System identification and control of robot manipulator based on fuzzy adaptive differential evolution algorithm. Adv Eng Softw. 2014;78:60–6. doi: 10.1016/j.advengsoft.2014.08.009

[74] Craig J. Introduction to robotics: mechanics and control, 3rd edn. Pearson Education; 2009.

[75] Serway RA, Jewett JW. Physics for scientists and engineers. Cengage Learning; 2018.

[76] Choi B, Kang D. Modeling and simulation of discrete event systems. John Wiley & Sons; 2013.

[77] Wu J, Wang J, You Z. An overview of dynamic parameter identification of robots. Robot Comput-Integr Manuf. 2010;26(5):414–9. doi: 10.1016/j.rcim.2010.03.013

[78] Gautier M. Optimal motion planning for robot’s inertial parameters identification. In: Proceedings of the 31st IEEE Conference on Decision Control. IEEE; 1992, pp. 70–3.

[79] Åström KJ, Eykhoff P. System identification—a survey. Automatica. 1971;7(2):123–62. doi: 10.1016/0005-1098(71)90059-8

[80] Miettinen K. Nonlinear multiobjective optimization. Springer Science & Business Media; 2012.

[81] Osyczka A, Kundu S. A new method to solve generalized multicriteria optimization problems using the simple genetic algorithm. Struct Optim. 1995;10(2):94–9. doi: 10.1007/bf01743536

[82] Betts JT. Practical methods for optimal control and estimation using nonlinear programming. SIAM; 2010.

[83] Coello CAC, Lamont GB, Van Veldhuizen DA. Evolutionary algorithms for solving multi-objective problems, vol. 5. Springer; 2007.

[84] Talbi E-G. Metaheuristics: from design to implementation, vol. 74. John Wiley & Sons; 2009.

[85] Ahmad MF, Isa NAM, Lim WH, Ang KM. Differential evolution: a recent review based on state-of-the-art works. Alex Eng J. 2022;61(5):3831–72. doi: 10.1016/j.aej.2021.09.013

[86] Shami TM, El-Saleh AA, Alswaitti M, Al-Tashi Q, Summakieh MA, Mirjalili S. Particle swarm optimization: a comprehensive survey. IEEE Access. 2022;10:10031–61. doi: 10.1109/access.2022.3142859

[87] Alhijawi B, Awajan A. Genetic algorithms: theory, genetic operators, solutions, and applications. Evol Intel. 2023;17(3):1245–56. doi: 10.1007/s12065-023-00822-6

[88] Wolpert D, Macready Wg. No free lunch theorems for search. SFI-TR-95-02-010. Santa Fe Institute; 1995.

[89] Derrac J, García S, Molina D, Herrera F. A practical tutorial on the use of nonparametric statistical tests as a methodology for comparing evolutionary and swarm intelligence algorithms. Swarm Evol Comput. 2011;1(1):3–18. doi: 10.1016/j.swevo.2011.02.002

[90] Huang C, Li Y, Yao X. A survey of automatic parameter tuning methods for metaheuristics. IEEE Trans Evol Comput. 2020;24(2):201–16. doi: 10.1109/tevc.2019.2921598

[91] Velasco-Carrau J, García-Nieto S, Salcedo JV, Bishop RH. Multi-objective optimization for wind estimation and aircraft model identification. J Guid Control Dyn. 2016;39(2):372–89. doi: 10.2514/1.g001294

[92] Isermann R, Schaffnit J, Sinsel S. Hardware-in-the-loop simulation for the design and testing of engine-control systems. Control Eng Practice. 1999;7(5):643–53. doi: 10.1016/s0967-0661(98)00205-6

[93] Swevers J, Verdonck W, De Schutter J. Dynamic model identification for industrial robots. IEEE Control Syst Mag. 2007;27(5):58–71.

[94] Benitez-Garcia SE, Villarreal-Cervantes MG. Event-triggered control for a three DoF manipulator robot. Enfoque UTE. 2018;9(4):33–44. doi: 10.29019/enfoqueute.v9n4.396

[95] Radkhah K, Kulic D, Croft E. Dynamic parameter identification for the CRS A460 robot. In: The 2007 IEEE/RSJ International Conference on Intelligent Robots and Systems. IEEE; 2007, pp. 3842–7.

[96] Krishnan R. Electric motor drives: modeling, analysis, and control. Prentice Hall; 2001.

[97] Ogata K, Yang Y. Modern control engineering. Upper Saddle River, NJ: Prentice Hall; 2010.

[98] Deb K, Kalyanmoy D. Multi-objective optimization using evolutionary algorithms. John Wiley & Sons; 2001.

[99] Worden K, Barthorpe RJ, Cross EJ, Dervilis N, Holmes GR, Manson G, et al. On evolutionary system identification with applications to nonlinear benchmarks. Mech Syst Signal Process. 2018;112:194–232. doi: 10.1016/j.ymssp.2018.04.001

[100] Sampson J. Adaptation in natural and artificial systems. John H. Holland; 1976.

[101] Epitropakis MG, Tasoulis DK, Pavlidis NG, Plagianakos VP, Vrahatis MN. Enhancing differential evolution utilizing proximity-based mutation operators. IEEE Trans Evol Comput. 2011;15(1):99–119. doi: 10.1109/tevc.2010.2083670

[102] Opara KR, Arabas J. Differential evolution: a survey of theoretical analyses. Swarm Evol Comput. 2019;44:546–58. doi: 10.1016/j.swevo.2018.06.010

[103] Rao S. Engineering optimization: theory and practice, 4th edn. John Wiley & Sons; 2009.

[104] Nocedal J, Wright SJ. Numerical optimization, 2nd edn. Springer; 2006.

[105] Williamson M. Series elastic actuators. Massachusetts Institute of Technology Artificial Intelligence Laboratory; 1995.

[106] Junior A, de Andrade R, Bento Filho A. Series elastic actuator: design, analysis and comparison. Recent Adv Robot Syst. 2016;1(3).

[107] Au SK, Weber J, Herr H. Biomechanical design of a powered ankle-foot prosthesis. In: 2007 IEEE 10th International Conference on Rehabilitation Robotics. IEEE; 2007, pp. 298–303. doi: 10.1109/icorr.2007.4428441

[108] Herr HM, Grabowski AM. Bionic ankle-foot prosthesis normalizes walking gait for persons with leg amputation. Proc Biol Sci. 2012;279(1728):457–64. doi: 10.1098/rspb.2011.1194 21752817 PMC3234569

[109] Grimmer M, Holgate M, Holgate R, Boehler A, Ward J, Hollander K, et al. A powered prosthetic ankle joint for walking and running. Biomed Eng Online. 2016;15(Suppl 3):141. doi: 10.1186/s12938-016-0286-7 28105953 PMC5249039

[110] Agarwal P, Deshpande AD. Series elastic actuators for small-scale robotic applications. J Mech Robot. 2017;9(3). doi: 10.1115/1.4035987

[111] Subramanim K, Sriraman A, Amirtham V, Rani A, Ali S, Perumal T. Review of current development of knee rehabilitation device using series elastic actuator (SEA). In: ICPER 2020 Proceedings of the 7th International Conference on Production, Energy and Reliability. Springer; 2022.

[112] Huo W, Mohammed S, Moreno JC, Amirat Y. Lower limb wearable robots for assistance and rehabilitation: a state of the art. IEEE Syst J. 2016;10(3):1068–81. doi: 10.1109/jsyst.2014.2351491

[113] Chiasson J. Modeling and high performance control of electric machines. John Wiley & Sons; 2005.

[114] Park Y, Paine N, Oh S. Development of force observer in series elastic actuator for dynamic control. IEEE Trans Ind Electron. 2018;65(3):2398–407. doi: 10.1109/tie.2017.2745457

[115] Palma-Huerta AA, Villarreal-Cervantes MG, Salgado-Ramos I de J. Desarrollo y análisis de la integración de la dinámica del motor en el modelo de un actuador elástico en serie con detección de fuerza de reacción.. ICBI. 2024;12:128–37. doi: 10.29057/icbi.v12iespecial4.13322

